# Boundary domain genes were recruited to suppress bract growth and promote branching in maize

**DOI:** 10.1126/sciadv.abm6835

**Published:** 2022-06-15

**Authors:** Yuguo Xiao, Jinyan Guo, Zhaobin Dong, Annis Richardson, Erin Patterson, Sidney Mangrum, Seth Bybee, Edoardo Bertolini, Madelaine Bartlett, George Chuck, Andrea L. Eveland, Michael J. Scanlon, Clinton Whipple

**Affiliations:** 1 Department of Biology, Brigham Young University, 4102 LSB, Provo, UT 84602, USA.; 2 Donald Danforth Plant Science Center, St. Louis, MO 63132, USA.; 3 Plant Gene Expression Center, Albany, CA 94710, USA.; 4 Institute of Molecular Plant Sciences, School of Biological Sciences, University of Edinburgh, Edinburgh, EH9 3BF, Scotland, UK.; 5 Biology Department, University of Massachusetts Amherst, Amherst, MA 01003, USA.; 6 Plant Biology Section, School of Integrative Plant Science, Cornell University, Ithaca, NY 14853, USA.

## Abstract

Grass inflorescence development is diverse and complex and involves sophisticated but poorly understood interactions of genes regulating branch determinacy and leaf growth. Here, we use a combination of transcript profiling and genetic and phylogenetic analyses to investigate *tasselsheath1* (*tsh1*) and *tsh4*, two maize genes that simultaneously suppress inflorescence leaf growth and promote branching. We identify a regulatory network of inflorescence leaf suppression that involves the phase change gene *tsh4* upstream of *tsh1* and the ligule identity gene *liguleless2* (*lg2*). We also find that a series of duplications in the *tsh1* gene lineage facilitated its shift from boundary domain in nongrasses to suppressed inflorescence leaves of grasses. Collectively, these results suggest that the boundary domain genes *tsh1* and *lg2* were recruited to inflorescence leaves where they suppress growth and regulate a nonautonomous signaling center that promotes inflorescence branching, an important component of yield in cereal grasses.

## Teaser

Grass inflorescence branching involves the integration of competing signals that regulate leaf and meristem growth.

## INTRODUCTION

The transition from vegetative to reproductive growth in plants is typically accompanied by marked morphological changes. Among these changes, leaf outgrowth, the dominant vegetative characteristic in most plants, is often highly reduced or completely suppressed. Leaves subtending reproductive structures (inflorescence branches or flowers) are called bracts, and some level of bract reduction or suppression is common but not universal in the angiosperms (*1*).

Similar to most grasses, maize suppresses a subset of inflorescence bracts, which are only visible as a small ridge during early stages of inflorescence development (*2*, *3*). While some inflorescence bracts (floret lemmas and spikelet glumes) are not suppressed in the grasses, all bracts subtending inflorescence branches and spikelets are suppressed. This selective bract suppression in grasses is morphologically distinct from other angiosperm lineages such as the Brassicaceae, where bracts subtending both flowers and inflorescence branches are generally suppressed (*4*). Positionally specific suppression of bracts is a morphological innovation of the grass family as close outgroups in Poales do not suppress bracts at any position in their inflorescence (*3*).

Analysis of several bract suppression mutants in maize has provided key insights into the molecular regulation of bract suppression in the grass family. These mutants include the *tassel sheath 1* to *5* (*tsh1* to *tsh5*) loci (*3*), of which two have been cloned. *tsh1* encodes a GATA domain zinc-finger transcription factor (*3*), while *tsh4* encodes a *SQUAMOSA PROMOTER BINDING PROTEIN* (*SBP*) transcription factor (*5*). The dominant *Cg1* also displays derepressed bracts and encodes a microRNA that targets *tsh4* and related *SBP* family members (*6*). Two *SBP* genes, *unbranched2* and *unbranched3*, are closely related to and function redundantly with *tsh4* to regulate bract suppression and inflorescence branching (*7*). Other *tsh* loci have not yet been cloned, but it is intriguing that genes from at least two unrelated transcription factor families are required for bract suppression in maize, suggesting that a complex transcriptional network for bract suppression evolved in the grass family.

Bract suppression in eudicots and grasses is likely controlled by distinct genes. In *Arabidopsis thaliana* (arabidopsis), bract suppressing genes include *LEAFY* (*8*) and *BLADE ON PETIOLE* (*BOP*) *1* and *BOP2* (*9*, *10*). Of these, *LFY* plays a major role, which appears to be conserved in eudicot lineages that independently evolved bract suppression, including Solanaceae and Fabaceae (*11*–*13*). However, loss-of-function mutants for orthologs of *LFY* and other eudicot bract suppression genes show no bract growth defects in the grasses (*14*–*16*). Similarly, grass bract suppression genes have no bract suppression role in arabidopsis. While *tsh4* and orthologous *SBP* genes have a conserved phase transition function in both eudicots (*17*) and monocots (*5*, *7*), *SBP-like 9* (*SPL9*) and *SPL15* genes, the arabidopsis orthologs of *tsh4*/*ub2*/*ub3*, do not influence bract suppression (*18*). A comparison of *tsh1* function across eudicots and grasses reveals even more divergence in their bract suppression pathways. Knockouts of the arabidopsis *tsh1* orthologs *HANABA TARANU* (*HAN*), *HAN-like1*, and *HAN-like2* show defects in floral organ initiation and separation, and embryo patterning consistent with a boundary domain function (*19*–*21*). These boundary phenotypes are not evident in *tsh1* mutants, indicating a substantial shift in *HAN* versus *tsh1* function at some point in angiosperm evolution. Why grasses evolved a novel and complex bract suppression network that is morphologically targeted to only a subset of inflorescence branching events is not clear.

While the developmental and evolutionary role of bract suppression is still an open question, a proposed explanation is that the emerging bract competes with the adjacent meristem for cells and growth factors. Bract suppression, in this interpretation, diverts limited growth resources away from leaves and toward meristem growth and branching after the floral transition (*22*–*25*). In support of this hypothesis, derepressed bract growth is correlated with reduced branching in maize *tsh1* and *tsh4* mutants (*3*, *5*). This correlation is not complete, however, as *tsh* mutants will often form bracts without affecting the determinacy of their adjacent meristem (*3*). Conversely, *ub2* and *ub3* affect branching but not bract growth, despite their localization to the bract primordium and redundant function when combined with *tsh4* to suppress bracts (*7*). The partial decoupling of bract growth from branch suppression raises the possibility that the suppressed bract and the meristem it subtends interact in a manner beyond mere competition for resources. One possibility is that *tsh1* and *tsh4* genes are involved in regulation of grass branch architecture as part of a suppressed bract signaling center that nonautonomously regulates the determinacy of the branch meristem (*26*).

Considering the importance of meristem determinacy to inflorescence architecture and yield traits in domesticated cereals, we sought to better understand the contribution of bract suppression to inflorescence development and the bract transcriptional networks regulated by *tsh1* and *tsh4*. Here, we report our investigation of genetic interactions and transcriptional changes associated with these bract suppression mutants. We identify a core transcriptional network involving *tsh1*, *tsh4*, and the boundary domain gene *liguleless2* (*lg2*), which jointly regulate both bract suppression and inflorescence branch meristem determinacy. We detect a series of duplications in the grass *tsh1* gene lineage that preceded the recruitment of this boundary domain gene to the suppressed bract. Our results suggest that the phase change regulator *tsh4* recruited *tsh1* and *lg2* to a targeted role in inflorescence development and that bract suppression indirectly resulted from recruiting these boundary domain genes to form a novel signaling center that promotes branch meristem indeterminacy in grasses.

## RESULTS

### *tsh4* acts synergistically with *tsh1* to regulate bract suppression and branch meristem indeterminacy

In an ongoing effort to characterize additional *tsh* loci, we identified a novel allele of *tsh4* (described previously as *tsh2*) containing a *Mutator* transposon insertion in the first intron, which we designated *tsh4-rm* (fig. S1, A and C). In a separate screen for genetic modifiers of the weak *tsh1-2* allele, we isolated a semidominant *tsh1* enhancer that was also allelic to *tsh4* (tsh4-ent*355; fig. S1, B and C), which segregated as a single recessive locus in the absence of *tsh1-2*. In addition, we identified a large deletion paired with a *Mu* transposon insertion as the causative lesion in the *tsh1-ref* allele (fig. S1D).

**Fig. 1.**
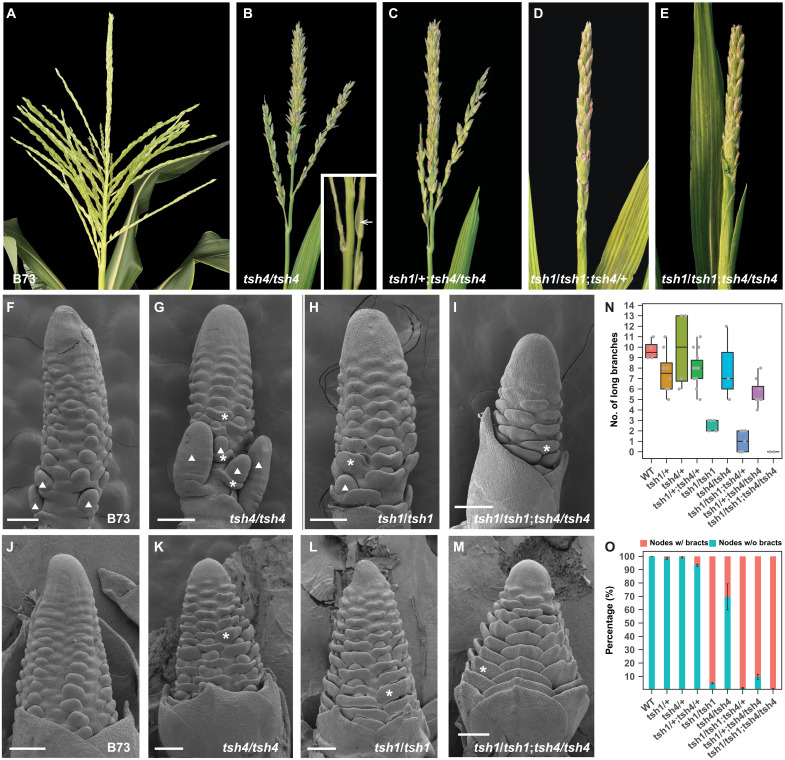
*tsh1* and *tsh4* act synergistically to regulate bract suppression and inflorescence branching. (**A** to **E**) Tassel phenotype of plants in *tsh1-ref/+*: *tsh4-rm/*+ (B73) segregating population showing progressive enhancement of *tsh4* (B) as *tsh1* function is progressively removed. B73, wild-type tassel control (A). (**F** to **M**) Scanning electron microscopy of tassel (F to I) and ear (J to M) inflorescence primordia of wild-type B73 (F and J), *tsh4* (G and K), *tsh1* (H and L), and *tsh1 tsh4* double mutant (I and M). (**N** and **O**) Quantification of branching (N) and bract (O) phenotype in all segregating genotypes. In (N), the colored boxes define the upper or lower quartile, with horizontal lines designating the median, and the gray dots represent individual data points. Arrow in (B) designates a bract, as do asterisks (*) in (F) to (M). Arrowheads in (F) to (M) indicate long tassel branches. Scale bars, 200 μm.

The marked enhancement of the weak *tsh1-2* phenotype by tsh4-ent*355 suggests a synergistic interaction. To more fully investigate the nature of this interaction, we measured both tassel branching and bract suppression in an F2 population segregating *tsh4-rm* and *tsh1-ref*, each introgressed ≥5× to the reference B73 genetic background. Introgression of *tsh4-rm* into B73 notably suppressed the phenotype (compare fig. S1A with Fig. 4D), indicating that natural modifiers in B73 ameliorate the phenotypic severity of *tsh4* mutants. Nevertheless, *tsh4* and *tsh1* showed a consistent and synergistic interaction. As shown in Fig. 1 and Table 1, *tsh1 tsh4* double mutant tassels produce no long tassel branches and, compared to *tsh1* and *tsh4* single mutants, *tsh1 tsh4* double mutant tassels also have a nonadditive increase in the percentage of solitary spikelets, empty nodes, and nodes with subtending derepressed bracts. The phenotypic enhancement was not limited to the double mutant, as *tsh1*/*+*; *tsh4*/*tsh4* and *tsh1*/*tsh1*; *tsh4*/*+* individuals had fewer branches and more bract growth than *tsh1* or *tsh4* single mutants.

**Table 1.** Phenotypic characterization of *tsh1*/*+*; *tsh4*/+ segregating population. A *tsh1-ref*/+, *tsh4-rm*/+ segregating population containing 57 plants was grown in Spanish Fork, UT in 2018 and PCR-genotyped. Mature tassels from all genotyped plants were inspected manually for branch and bract growth. In this table, we report the mean value for each genotype with the SEM in parentheses. Statistical significance was determined by one-way analysis of variance (significance level = 0.05) followed by post hoc comparisons to determine whether statistically significant differences exist between the *tsh1* single mutant (*tsh1*/*tsh1* homozygote) and the *tsh1 tsh4* mutants (*tsh1*/*tsh1*; *tsh4*/+ mutant or *tsh1*/*tsh1*; *tsh4*/*tsh4* mutant) or between *tsh4* single mutant (*tsh4*/*tsh4* homozygote) and the *tsh1 tsh4* mutants (*tsh1*/+; *tsh4*/*tsh4* mutant or *tsh1*/*tsh1*; *tsh4*/*tsh4* mutant) by using the Fisher’s least significant difference method. Significant difference, **P < 0.01; ***P < 0.001; ns, not significant.

**Genotype**	**No. of long branches**	**% Nodes with long branches**	**% Nodes with paired spikelets**	**% Nodes with solitary spikelets**	**% Empty nodes**	**% Nodes with subtending bracts**
*WT*	9.750 (0.479)	0.040 (0.005)	0.907 (0.024)	0.053 (0.019)	0.000 (0.000)	0.000 (0.000)
*tsh1/+*	7.625 (0.730)	0.030 (0.002)	0.941 (0.012)	0.029 (0.010)	0.000 (0.000)	0.015 (0.008)
*tsh4/+*	9.750 (1.887)	0.039 (0.004)	0.904 (0.008)	0.057 (0.007)	0.000 (0.000)	0.008 (0.005)
*tsh1/+;tsh4/+*	8.000 (0.432)	0.032 (0.002)	0.907 (0.008)	0.0061 (0.006)	0.001 (0.000)	0.067 (0.008)
*tsh4/tsh4*	8.000 (2.082)	0.030 (0.005)	0.792 (0.055)	0.170 (0.050)	0.009 (0.003)	0.304 (0.098)
*tsh1/+;tsh4/tsh4*	5.625 (0.460)^ns^	0.032 (0.0003)^ns^	0.463 (0.037)^***^	0.498 (0.038)^***^	0.007 (0.002)^ns^	0.903 (0.019)^***^
*tsh1/tsh1*	2.600 (0.245)	0.021 (0.002)	0.753 (0.025)	0.216 (0.024)	0.010 (0.003)	0.952 (0.004)
*tsh1/tsh1 tsh4/+*	1.000 (0.333)^ns^	0.010 (0.003)^**^	0.591 (0.033)^***^	0.360 (0.032)^***^	0.040 (0.007)^***^	0.987 (0.004)^ns^
*tsh1/tsh1;tsh4/tsh4*	0.000 (0.000)^***^	0.000 (0.000)^**^	0.289 (0.036)^***^	0.543 (0.043)^***^	0.168 (0.007)^***^	0.994 (0.006)^***^

To investigate the early ontogeny of the branching and bract growth defects, we examined tassel and ear primordia of *tsh1* and *tsh4* single and double mutants by scanning electron microscopy (Fig. 1, F to M). While most nodes were associated with a derepressed bract in *tsh1*, bract growth was more pronounced in the double mutant, particularly in the ear. While the axillary meristems were clearly subtended by derepressed bracts in *tsh1* and *tsh4* single mutants, their meristems initiated at later nodes compared to B73. In addition, *tsh1 tsh4* double mutants lack any obvious axillary meristems at early stages, although older nodes may have meristems obscured by the large bracts (Fig. 1M). These results confirm that *tsh1* and *tsh4* act redundantly to suppress bract growth and promote meristem initiation.

### *tsh1* and *tsh4* regulate diverse pathways involved in meristem and leaf development, hormone signaling, and boundary domains

**Fig. 2.**
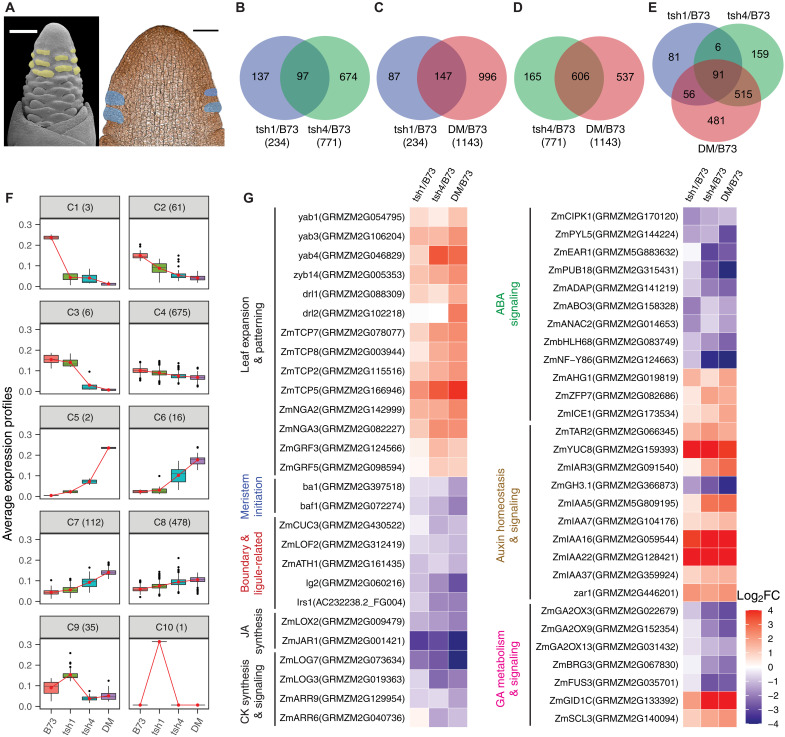
Transcript profiling of LM suppressed and growing ear bract primordia. (**A**) Scanning electron microscopy (left) and thin section (right) of young ear primordium indicating the cells targeted for laser capture (yellow and blue, respectively). Scale bars, 200 μm (left) or 100 μm (right). (**B** to **E**) Venn diagram of common and unique differentially regulated bract genes in *tsh1*, *tsh4* single and *tsh1 tsh4* double mutants (DM). (**F**) Ten coexpression clusters (C1 to C10) were identified from the 1389 genes that were differentially expressed between B73 and the *tsh* mutants. Connected red lines correspond to the mean expression profiles for each cluster. Boxes define the upper or lower quartile, and dots outside the bars indicate outliers. (**G**) Genes with well-documented function in leaf expansion and patterning, meristem initiation, boundary and ligule establishment, and hormone metabolism/signaling were differentially expressed in *tsh* mutants compared to that in the wild type. FC, fold change.

Transcripts of both *tsh1* and *tsh4* are localized to the bract primordium from the earliest stages of bract initiation (*3*, *5*, *6*). However, the molecular processes regulated by these genes within this very narrow domain are unclear. To identify transcriptional changes associated with *tsh1-* and *tsh4*-mediated bract suppression, we generated RNA sequencing (RNA-seq) transcriptomes of laser-microdissected (LM) bract primordia of the wild-type (B73), *tsh1*, *tsh4*, and *tsh1 tsh4* double mutants. Specifically, we collected cells from ear bract primordia where the bract ridge, but not the adjacent meristem, is visible (Fig. 2A). Principal components analyses (PCAs) confirmed that the biological replicates were highly correlated within genotypes (fig. S2). In addition, *tsh1* and *tsh4* transcript levels were significantly down-regulated in their respective mutants (fig. S3). To confirm the tissue specificity of our LM, we investigated the known suppressed bract marker *zea yabby15* (*zyb15*) (*3*), as well as the meristem-specific *Knotted1* (*Kn1*), which is strongly down-regulated in the suppressed bract (*27*). As expected, there was significant enrichment of *zyb15* and reduction of *Kn1* compared to their expression in LM shoot apical meristem tissue (fig. S4). Thus, our transcriptomes are reliable and will likely uncover transcriptional changes associated with bract suppression downstream of *tsh1* and *tsh4*.

In total, 31.8% (20,168) of maize annotated genes (AGPv3_5b+) were expressed in at least one sample (table S1). Of these, 6.9% (1389) were differentially expressed genes (DEGs) between B73 and at least one of the mutants (Fig. 2, B to E, and tables S2 to S5). Over three times more genes were differentially expressed in *tsh4* (771) compared to *tsh1* (234), suggesting that *tsh1* regulates a narrower set of downstream genes (Fig. 2B). DEGs in the *tsh1 tsh4* double mutant included not only most of the genes that were differentially expressed in *tsh1* and *tsh4* individually (60 and 78%, respectively) but also an additional 481 DEGs not present in either single mutant, consistent with the synergistic phenotype of *tsh1 tsh4* (Fig. 2, C to E). The PCA was largely consistent with these conclusions, as PC1 (63% of variance) is mostly explained by *tsh4*, while PC2 (17% of variance) is explained by *tsh1*, indicating partially nonoverlapping roles for *tsh1* and *tsh4*, but with more notable impact from *tsh4* (fig. S2). While *tsh1* and *tsh4* have distinct DEG profiles, a significantly larger portion of *tsh1* DEGs were shared with *tsh4* than vice versa (41% versus 12%) (Fig. 2B). This raises the possibility that *tsh1* functions downstream of *tsh4*. Therefore, we examined expression of *tsh1* in *tsh4* single mutants and vice versa. While *tsh4* expression in *tsh1* single mutants is similar to B73 controls, *tsh1* is significantly decreased in *tsh4* mutants (fig. S3), consistent with *tsh4* functioning upstream of *tsh1*.

To gain further insight into the molecular processes associated with bract suppression, we used a combination of *K*-means clustering and Gene Ontology (GO) classification enrichment analysis on DEGs in at least one *tsh* mutant. *K*-means analysis identified 10 unique clusters of coexpressed genes across the different genotypes (Fig. 2F and table S6, A to E). Clusters 1 to 4 showed similar patterns of decreased expression in the mutants; genes in these clusters likely function downstream of *tsh1* and *tsh4* to repress bract growth. Clusters 5 to 8 showed the opposite trend, with increased expression in the mutants; genes in these clusters likely promote bract outgrowth. A common trend observed in clusters 1 to 8 was a larger response in *tsh4* mutants compared to *tsh1*, with the strongest response in the double mutant, consistent with *tsh4* acting upstream of *tsh1*. In contrast, clusters 9 and 10 include a small set of genes that were up-regulated only in *tsh1*. We performed GO enrichment analysis for genes in clusters 1 to 4 and 5 to 8 (fig. S5). Overall, these clusters are enriched for genes involved in the regulation of gene expression, organ developmental processes (leaf, root, flower, inflorescence, meristem), cell growth and differentiation, and hormone metabolism and signaling. Some enriched GO categories were exclusive to the upward versus downward trending expression in clusters 1 to 4 versus 5 to 8. In particular, genes involved in inflorescence development, gibberellin metabolic process, response to abscisic acid (ABA), response to ethylene, extracellular matrix assembly, and dormancy were unique to clusters 1 to 4, while genes involved in leaf morphogenesis, auxin metabolic process, auxin transport, and auxin-activated signaling pathways were only enriched in clusters 5 to 8. Clusters 9 and 10 contain only 36 genes with no significantly enriched GO terms.

A closer look at individual DEGs revealed several genes with known roles in leaf expansion and patterning as well as branch meristem initiation and determinacy. These results were largely expected based on the bract growth and branch suppression phenotypes of *tsh1* and *tsh4*. In addition to these genes, we were particularly interested in genes involved in hormone metabolism/signaling and boundary formation given the known function of *HANABA TARANU* (*HAN*, the arabidopsis *tsh1* ortholog) as a boundary gene that regulates hormone dynamics (*19*–*21*).

#### Leaf expansion and patterning

Multiple transcription factor families with well-documented roles in leaf development and patterning were differentially regulated in *tsh* mutants, consistent with the bract outgrowth phenotype of these mutants (Fig. 2G). This included up-regulation of multiple *YABBY* transcription factors (*yab1*, *yab3*, *zyb14*, *drl1*, and *drl2*), which have critical roles in leaf expansion (*28*, *29*). In addition, *TEOSINTE BRANCHED1*, *CYCLOIDEA*, and *PCF1* (*TCP*) transcription factors including both class I (*ZmTCP7* and *ZmTCP8*) and class II TCP-CIN (*ZmTCP2* and *ZmTCP5*) orthologs were up-regulated in *tsh.* While the function of class I TCPs is poorly understood, they are thought to regulate cell division (*30*). Class II TCP orthologs, however, have a well-documented role in leaf development (*31*) and interact with *NGATHA* (*NGA*) genes to promote cell differentiation and leaf expansion (*32*)*. NGA2* and *NGA3* are also up-regulated in *tsh* bracts. Last, two *GROWTH-REGULATING FACTOR* transcription factors (*GRF3* and *GRF5*) are up-regulated. These genes regulate cell proliferation in Arabidopsis (*33*) and are enriched in the actively dividing regions of the maize leaf (*34*), consistent with a role in early leaf expansion.

#### Meristem initiation

Inflorescence branching is controlled by genes that regulate axillary meristem initiation and determinacy. In maize, the transcription factors *barren stalk1* (*ba1*) and *barren stalk fastigiate1* (*baf1*) are required for axillary meristem initiation in the inflorescence (*35*, *36*). Both genes are significantly down-regulated in *tsh* mutants (Fig. 2G), consistent with the reduced and delayed branch meristem initiation in *tsh* mutants. *ba1* and *baf1* are not expressed in the suppressed bract, but in an adjacent boundary domain adaxial to the axillary meristem. It is possible that adjacent cells expressing *ba1/baf1* were sampled inadvertently from the margins of captured bract cells during laser microdissection.

#### Hormone homeostasis and signaling

Lateral organ growth is regulated by a complex interplay of hormone signaling. In light of this, it is not surprising to find a number of hormone signaling genes that were differentially regulated. Specifically, our data consistently indicate that ABA, auxin, and gibberellic acid (GA) signaling were altered.

ABA signaling components including maize orthologs of *CBL-INTERACTING PROTEIN KINASE 1* (*CIPK1*), *PYRABACTIN RESISTANCE 1-LIKE 5* (*PYL5*), *ENHANCER OF ABA CO-RECEPTOR 2* (*EAR1*), *PLANT U-BOX 18* (*PUB18*), *ARIA-INTERACTING DOUBLE AP2 DOMAIN PROTEIN* (*ADAP*), *ABA OVERLY SENSITIVE MUTANT 3* (*ABO3*), *ARABIDOPSIS NAC DOMAIN CONTAINING PROTEIN 2* (*ANAC2*), *BASIC HELIX-LOOP-HELIX DNA-BINDING FAMILY PROTEIN* (*bHLH68*), and *NUCLEAR FACTOR Y*, *SUBUNIT B6* (*NF-YB6*) were down-regulated in *tsh* mutants (Fig. 2G), suggesting that *tsh1* and *tsh4* promote ABA signaling in the suppressed bract. Consistent with this, the orthologs of three negative regulators of ABA signaling, including *ABA-HYPERSENSITIVE GERMINATION 1* (*AHG1*), *ZINC FINGER PROTEIN 7* (*ZFP7*) (*37*), and *INDUCER OF CBF EXPRESSION 1* (*ICE1*) (*38*), were up-regulated in *tsh* mutants (Fig. 2G). As ABA is associated with dormancy and growth inhibition in multiple developmental contexts (*39*), *tsh1/tsh4* may promote ABA signaling to inhibit bract outgrowth.

Auxin is another crucial regulator of lateral organ initiation and outgrowth (*40*). The auxin biosynthesis genes *TRYPTOPHAN AMINOTRANSFERASE RELATED 2* (*TAR2*) and *YUCCA 8* (*YUC8*) and the auxin conjugate hydrolase *IAA-ALANINE RESISTANT 3* (*IAR3*) were up-regulated in *tsh* mutants, whereas the auxin inactivation gene indole-3-acetic acid (IAA) amido synthetase *GH3.1* was down-regulated compared to wild type (Fig. 2G). This suggests that *tsh1* and *tsh4* may inhibit auxin production in the suppressed bract primordium. Consistent with this, auxin-responsive Aux/IAA family transcription factors including *IAA5*, *IAA7*, *IAA16*, *IAA22*, and *IAA37* were up-regulated in *tsh* mutants. Similarly, the auxin-inducible gene *zar1*, a positive regulator of cell proliferation and lateral organ size (*41*), was up-regulated, consistent with the bract outgrowth phenotype in *tsh* mutants.

GA promotes organ growth by inducing cell division and elongation (*42*). We found that the GA inactivation enzymes *ZmGA2OX3*, *ZmGA2OX9*, and *ZmGA2OX13* and GA response inhibitors *BOI-RELATED GENE 3* (*BRG3*) (*43*) and *FUSCA3* (*FUS3*) (*44*) were down-regulated in *tsh* mutants (Fig. 2G). Conversely, orthologs of a GA receptor *GA INSENSITIVE DWARF1C* (*GID1C*) and the positive regulator of GA signaling *SCARECROW-LIKE 3* (*SCL3*) (*45*) were up-regulated. Together, these data indicate that *tsh1* and *tsh4* potentially inhibit GA signaling to suppress bract growth.

In addition to ABA, auxin, and GA, several genes involved in the metabolism and/or signaling of jasmonic acid (JA) and cytokinin (CK) were differentially expressed between wild-type and *tsh* bracts (Fig. 2G). These included orthologs of the JA biosynthesis genes *LIPOXYGENASE 2* (*LOX2*) and *JASMONATE RESISTANT 1* (*JAR1*), which were down-regulated in *tsh* mutants. CK-activating enzymes [*LONELY GUY 3* (*LOG3*) and *LOG7*] and CK signaling components [*TYPE-A RESPONSE REGULATOR 6* (*ARR6*) and *ARR9*] were down-regulated in *tsh* mutants, suggesting attenuated CK biosynthesis and signaling in the *tsh* bracts. Together, our transcriptomic analysis suggests that *tsh1* and *tsh4* promote ABA, JA, and CK signals while attenuating auxin and GA signals in the bract primordium.

#### Boundary domain and ligule-associated genes

Boundary domain genes were first described in eudicots where they separate and promote morphogenesis of determinate lateral organs from indeterminate meristems (*46*). Less is known about boundary formation in the monocots, and grasses in particular appear to have a novel boundary in the leaf that separates proximal sheath from distal blade compartments (*47*). The morphology of this boundary region is complex and composed of both ligule and auricle tissues, but we will use the term “ligular boundary” to refer to the entire boundary region. Mutants defective in the ligular boundary also have defects in tassel branching (*48*–*50*) similar to *tsh* mutants. Furthermore, the rice *tsh1* ortholog *NECKLEAF1* is expressed in the ligular boundary (*51*), and *tsh1* expression was shown to be enriched in the ligular boundary compared to adjacent blade and sheath tissue (*52*). While there is no obvious ligule phenotype on vegetative leaves of *tsh* mutants, we did notice ligule-auricle disruptions on the flag leaf of *tsh1-2* mutants (fig. S7C). Considering these correlations between boundary genes, ligules, and tassel branching, we were curious whether boundary or ligule genes were differentially regulated in *tsh* mutants.

We found several genes with documented boundary domain functions in arabidopsis including orthologs of *CUP SHAPED COTYLEDON3* (*CUC3*) (*53*), *LATERAL ORGAN FUSION2* (*LOF2*) (*54*), and *ARABIDOPSIS THALIANA HOMEOBOX GENE1* (*ATH1*) (*55*), all of which were down-regulated in the *tsh* mutants (Fig. 2G).

In addition to canonical boundary domain genes, we found that *lg2* and *liguleless-related sequence1* (*lrs1*), a paralog of *lg2*, were down-regulated in *tsh* mutants (Fig. 2G). Considering the apparent connections between bracts and the ligular boundary discussed above, we were curious whether transcriptional changes associated with ligular boundary were similarly present in our dataset. To investigate this, we compared our bract transcriptome with a published LM expression profile of cells early in ligule specification (*52*). Compared to neighboring blade and sheath cells, the emergent ligule was enriched for 619 genes (*52*). Among these 619 DEGs, a significant portion (141 genes, 22.8%, hypergeometric test *P* value = 3.209 × 10^−99^) were also differentially expressed between wild-type and *tsh* bract primordia (fig. S6 and table S7). The same study also identified 96 DEGs between wild type and *liguleless1* (*lg1*), another gene required for ligule development (*56*). A significant proportion of these (24 genes, 25%, hypergeometric test *P* value = 1.967 × 10^−15^) were also differentially expressed in *tsh* mutants (fig. S6). In addition, 21 (87.5%) of these 24 shared DEGs exhibited a similar trend of expression change in *lg1* and *tsh* mutants (table S8). Together, these results confirm that many transcriptomic changes associated with ligule determination are also associated with bract suppression.

### TSH4 binds regulatory DNA of *tsh1* and *lg2*

**Fig. 3.**
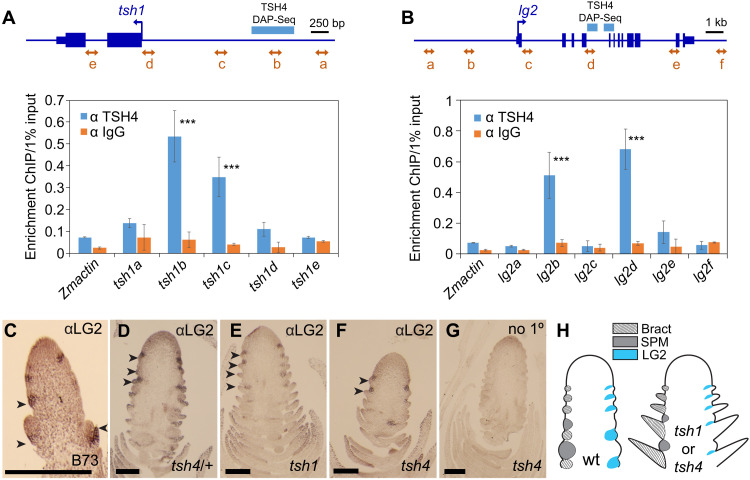
TSH4 binds promoters of *tsh1* and *lg2* and is necessary for suppressed bract expression of *lg2*. (**A**) Anti-TSH4 ChIP using primers designed to five locations (arrows a to e) in the *tsh1* genomic region. Bar graph shows enrichment of an anti-TSH4 ChIP compared to an anti-IgG control. Significant enrichment was found for promoter regions *tsh1b* and *tsh1c*. (**B**) Anti-TSH4 ChIP as in (A), using primers designed to six regions (a to f) of the *lg2* genomic region. Significant enrichment was observed for the promoter (*lg2b*) and the fourth intron (*lg2d*). TSH4 DAP-seq peak regions as identified in (*57*). (**C** to **F**) Immunolocalization of LG2 on a wild-type (B73) tassel primordium (C) and ear primordia of *tsh4/+* (D), *tsh1* (E), and *tsh4* (F). (**G**) Immunolocalization control without primary anti-LG2 antibody shows that the bract and boundary domain localization in (C) to (F) is specific to anti-LG2. (**H**) Summary of LG2 localization in the wild-type (wt) (left) and *tsh1* or *tsh4* mutants (right). SPM, spikelet pair meristem. Arrowheads in (C) to (F) indicate bract primordia. Scale bars, 0.5 mm (C to G). ***P < 0.001, two-tailed Student’s *t* test.

The down-regulation of *tsh1* in *tsh4* mutants suggests that *tsh4* is upstream of *tsh1* in a bract suppression network. To test whether this interaction is direct, we used a previously developed TSH4 antibody (*5*) to perform chromatin immunoprecipitation (ChIP). The TSH4 antibody was used to precipitate chromatin from ≤5-mm ear primordia, and enrichment was quantitated by quantitative polymerase chain reaction (qPCR) amplification of five regions across the *tsh1* gene (Fig. 3A). Compared with chromatin purified by immunoglobulin G (IgG) negative control, strong enrichment was observed for two adjacent regions (b and c) in the *tsh1* promoter approximately 1.5 kb upstream of the transcription start site. In addition, we found that these two ChIP-enriched regions overlap a region bound by TSH4 in a TSH4 DAP-seq (DNA affinity purification sequencing) dataset (*57*), demonstrating that *tsh1* is a direct binding target of TSH4 in vivo. While *tsh1* levels are strongly reduced in *tsh4* mutants, some residual expression suggests that additional factors are required to initiate *tsh1* in the suppressed bract, consistent with the notably milder bract suppression phenotype of *tsh4* compared to *tsh1*.

Among the genes differentially regulated by *tsh1* and *tsh4* is *lg2*, a transcription factor required for ligule development (*58*). Considering the reduced branching of *lg2* mutants (*48*), *lg2* may interact with *tsh1* and *tsh4* in their branch promotion roles. While *lg2* mutants were not originally described as having any bract suppression defects, we noticed that both tassels and ears of *lg2* mutants stochastically produce large bracts with low penetrance (fig. S7, A and B), further pointing to a connection between bract regulation and branch meristem determinacy.

We asked whether transcriptional regulation of *lg2* could be explained by direct binding of TSH4 to its promoter. Through ChIP qPCR across the *lg2* genic region, we identified two strong binding regions located in the proximal promoter and the fourth intron, respectively (Fig. 3B). Of these two apparent binding sites, the fourth intron was also bound by TSH4 in a TSH4 DAP-seq dataset (*57*). These results suggest that *lg2* is a transcriptionally modulated direct target of TSH4. Our results reveal that *tsh4* is a positive regulator of *tsh1* and *lg2.* In addition, since *lg2* is down-regulated in *tsh1* single mutants, *tsh1* appears to positively regulate *lg2* independent of and redundantly with *tsh4*.

To investigate the localization of LG2 during tassel development, we raised an antibody specific to LG2 (fig. S7C) and used this for immunolocalization at early stages of inflorescence development. We found that LG2 localizes to a broad domain that includes both the suppressed bract and the boundary between the suppressed bract and the adjacent meristem (Fig. 3, C and D). The localization of LG2 overlaps with the bract expression of *tsh1* and *tsh4* (*3*, *5*). Given the reduced *lg2* mRNA levels in both *tsh1* and *tsh4*, we hypothesized that these regulators of bract suppression are necessary to promote LG2 protein accumulation within the suppressed bract. LG2 expression is indeed reduced or absent from the suppressed bract region of *tsh1* and *tsh4* mutants, while largely maintained in the narrow boundary between the suppressed bract and the adjacent meristem (Fig. 3, E to G), confirming our LM RNA-seq and ChIP data that *lg2* is downstream of *tsh1* and *tsh4* within the suppressed bract.

### *lg2* interacts synergistically with *tsh1* and *tsh4* to regulate bract suppression and branch meristem determinacy

**Fig. 4.**
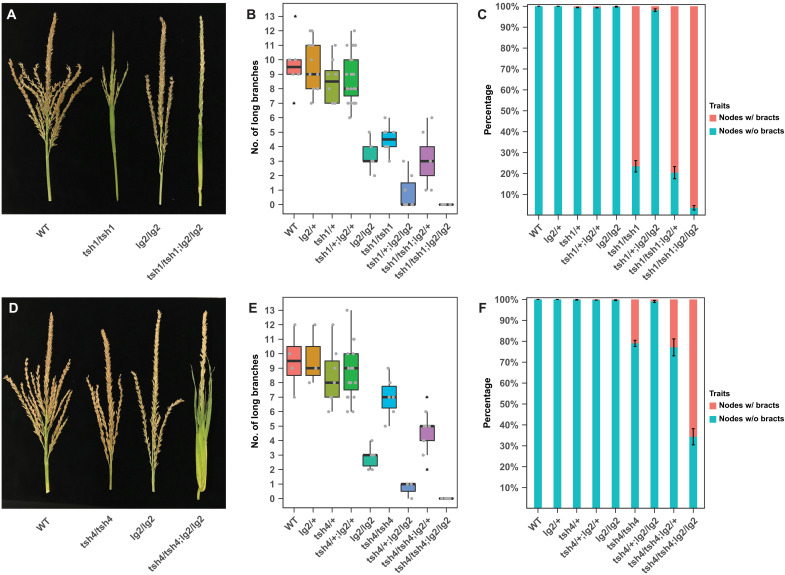
Synergistic *tsh1 lg2* and *tsh4 lg2* interactions promote bract growth and branch repression. (**A** to **C**) *tsh1 lg2* and (**D** to **F**) *tsh4 lg2* genetic interactions. Mature tassel phenotypes (A and D) show an enhancement of bract growth and reduced tassel branching in double mutants, which is further confirmed in a quantification of the number of long branches (B and E) and nodes (i.e., branching sites of the inflorescence) with bracts (C and F). In (B) and (E), the colored boxes define the upper or lower quartile with horizontal lines designating the median, gray dots represent the individual data points, and the black triangles indicate outliers. WT, wild type.

Our observation that *tsh1* and *tsh4* redundantly promote *lg2* in the suppressed bract raises the possibility that these factors cooperate in bract suppression and/or inflorescence branch meristem determinacy. To assess any genetic interaction of *lg2* with *tsh1* or *tsh4*, we generated *tsh1*/*+*; *lg2*/*+* and *tsh4*/*+*; *lg2*/*+* segregating populations using alleles introgressed into the B73 background and compared the tassel phenotype of each individual genotype in the populations (Fig. 4 and tables S9 and S10). Removing a copy of *lg2* (*lg2*/+) from a *tsh1* or a *tsh4* homozygous background significantly reduced long basal branches (Fi. 4, B and E). Similarly, removing a copy of *tsh1* (*tsh1*/+) or *tsh4* (*tsh4*/+) from a homozygous *lg2* background reduced long branches. Double mutants of *tsh1 lg2* and *tsh4 lg2* completely lacked long branches. Similar shifts of paired to solitary spikelets or nodes lacking any spikelet were observed for each of these genotypes (tables S9 and S10). While *lg2* only rarely produces tassel bracts, removing a copy of *tsh1* (*tsh1/+*) or *tsh4* (*tsh4/+*) from an *lg2* homozygous background resulted in consistent bract production, and the double mutants (both *tsh1 lg2* and *tsh4 lg2*) had a significant increase in tassel nodes with bracts (Fig. 4, C and F). These synergistic interactions are consistent with redundant and cooperative roles for *lg2* with both *tsh1* and *tsh4* in suppressing inflorescence bract growth and promoting branch meristem indeterminacy. We also noticed that while bracts often subtended branches in *tsh* mutants, reduced branches (solitary spikelets and empty nodes) were not always subtended by bracts (fig. S8 and tables S11 and S12), consistent with a role for *tsh1* and *tsh4* in promoting branch indeterminacy independent of their role in bract suppression. Furthermore, meristem determinacy defects were significantly more pronounced in the long branch zone compared to the central spike in both double mutant populations (fig. S9), suggesting a proximo-distal gradient of *tsh1*, *tsh4*, and *lg2* meristem determinacy activity. Overall, these genetic interactions further confirm that *lg2* functions in a common regulatory network with *tsh1* and *tsh4*, and underscore the importance of boundary domain genes in bract suppression and associated branch meristem determinacy.

### *tsh1* was likely recruited from an ancestral boundary domain function

**Fig. 5.**
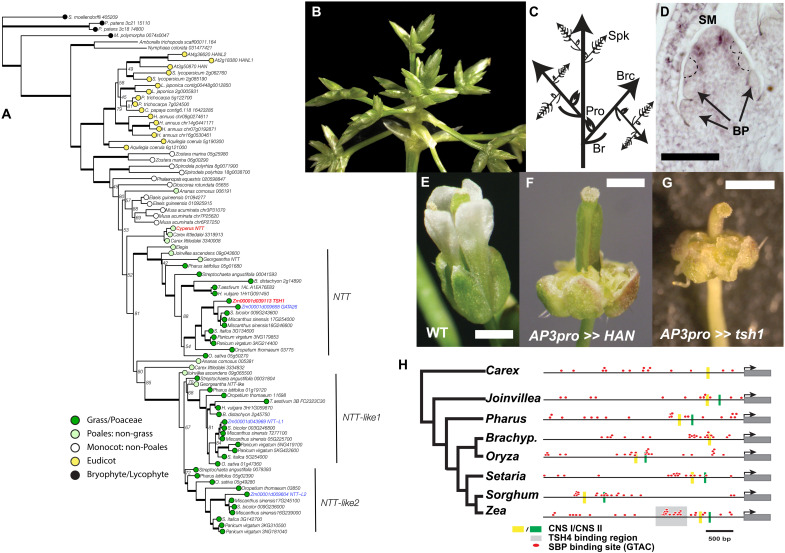
The NTT lineage in the grass family may result from neofunctionalization and maintains an ancestral boundary domain activity. (**A**) Phylogeny of the *HAN-NTT* gene family. (**B** and **C**) *Cyperus* inflorescence shows prominent bracts and prophylls associated with all inflorescence branching events. Br, bract; Brc, branch; Pro, prophyll; Spk, spikelet. (**D**) In situ localization of *Cyperus NTT* in inflorescence shows that *Cyperus NTT* expresses in the boundary domain separating the spikelet meristem from lateral bract primordia. (**E** to **G**) Phenotypes of arabidopsis florets with ectopic expression of *HAN* (F) or *Tsh1* (G) under the *AP3* promoter. (**H**) Distribution of putative SBP binding sites in 5′ promoters of *NTT* genes from grasses and close outgroups. CNS, conserved noncoding site; SM, spikelet meristem; BP, bract primordium. Dashed lines outline bract anlagen. Scale bars, 50 μm (D) and 1.0 mm (E to G).

The expression pattern and mutant phenotypes of *tsh1* and orthologous genes in the grasses (*3*, *59*) sharply contrast with arabidopsis *HAN* (*60*). One possible explanation for this divergence is that the boundary domain expression and function in arabidopsis is ancestral, and that the grass *NECKLEAF1*, *tsh1*, *THIRD OUTER GLUME* (*NTT*) clade evolved a novel expression and function related to bract suppression and branch promotion. Given the known duplications of the eudicot *HAN-like* and grass *NTT* genes (*3*, *19*), such neofunctionalization is a possibility. As a first step toward investigating the functional divergence of the *HAN-NTT* gene family, we reconstructed their phylogeny focusing in particular on the history of duplications in the grasses (Poaceae) and broader Poales (Fig. 5A). Our analysis revealed that the *HAN-NTT* subfamily of GATA domain transcription factors (i.e., those containing both a GATA and HAN domain) is present throughout the land plants, including the liverwort *Marchantia polymorpha* and the moss *Physcomitrium patens*. Within the eudicots, multiple duplications are apparent, and although our sampling was not sufficient to resolve the timing for each of these, none showed evidence of dating to deep nodes.

In the monocots, we identified a well-supported clade of Poales *NTT* genes. Within this Poales clade, we identified a duplication resulting in two distinct clades: *NTT* and *NTT-like1/2*. Members of the *NTT-like* clade are found in all Poales lineages sampled including *Joinvillea* and *Carex* (Cyperaceae) and *Ananas* (Bromeliaceae). However, the *NTT* clade includes no representative from *Ananas* or Cyperaceae, which are sister to the larger *NTT/NTT-like* clade, although the placement of these Cyperaceae and *Ananas* paralogs is poorly supported. While the precise timing remains uncertain, the duplication that created the *NTT* and *NTT-like* clades predates the origin of the grass family and may correspond to the sigma duplication event early in the Poales (*61*). Before the diversification of the grass family, there was a second duplication event creating the *NTT-like1* and *NTT-like2* clades. The paralogous *NTT-like1* and *NTT-like2* genes are present in all sampled grasses, and the timing of this duplication is consistent with the well-documented rho whole-genome duplication event (*61*). While our phylogeny does not show support for *Ananas* or Cyperaceae paralogs in the *NTT* lineages, given the known history of whole-genome duplications at the base of the grasses and Poales, it is more likely that a single duplication created paralogs of *NTT* and *NTT-like* in all Poales including *Ananas* and Cyperaceae rather than a more complicated series of duplication and loss, consistent with the poorly supported topology we recovered.

While *tsh1* and orthologous *NTT* genes in the grass family have a conserved role in bract suppression, the duplication that created the *NTT* lineage clearly predates the origin of bracts, raising the question of what this clade of genes did before they were involved in bract suppression. To infer the likely ancestral expression of the *NTT* lineage, we performed in situ hybridization using the likely *NTT* ortholog from *Cyperus.* The *Cyperus* inflorescence has prominent bracts and prophylls associated with all inflorescence branching events (Fig. 5, B and C). *Cyperus NTT* RNA was present in a distinct boundary domain separating the spikelet meristem from lateral bract primordia (Fig. 5D) but was not in bracts or other lateral organs. This boundary domain expression is similar to that reported for *HAN* in arabidopsis (*60*) and suggests that suppressed bract expression of *NTT* genes in the grass family is a neofunctionalization that arose after the duplication event that created the *NTT* lineage.

Neofunctionalization can involve changes to gene expression domains as well as to protein function. While grass *NTT* genes likely evolved a novel expression domain in the suppressed bract, it is not clear whether the protein function also diverged. We reasoned that if both HAN and TSH1 proteins maintain an ancestral boundary domain function to suppress organ growth (*46*, *47*), ectopic expression of either protein in young lateral primordia would suppress their growth. Consequently, we ectopically expressed TSH1 and HAN in lateral organs of arabidopsis (Fig. 5, E to G). To avoid the deleterious effects of suppressing all leaf growth, we used the arabidopsis *AP3* promoter:LhG4 fusion (p*AP3*) to drive expression of 10-OP:*HAN* and 10-OP:*tsh1* complementary DNA (cDNA) fusions just in petal and stamen lateral primordia. Both p*AP3>>HAN* and p*AP3>>tsh1* flowers lacked petals and stamens, consistent with our hypothesis that HAN and TSH1 have a conserved boundary domain function of inhibiting organ growth. Intriguingly, the overexpression phenotype was not limited to petal and stamen suppression but included growth of unorganized callus-like tissue in the same position of stamens and petals. This result was unexpected and suggests that *HAN* and *tsh1* are sufficient not only to inhibit growth but also to promote dedifferentiation and callus formation, which may be a result of the strong expression driven by the *AP3* promoter combined with the apparent hormone-modifying activity of *HAN* (*19*–*21*) and *tsh1* (this study).

The shift in *NTT* expression from boundary regions in a grass outgroup to the suppressed bract in the grasses likely results from grass *NTT* genes coming under the regulation of novel upstream factors. Since *tsh4* in maize and other grasses maintains an ancestral expression pattern in lateral organs (*5*, *62*, *63*), one possibility is that TSH4 binding to the *tsh1* promoter recruited this gene to a novel domain of inflorescence bracts. We examined 5 kb of 5′ promoter regions of *tsh1* and *NTT* genes in grasses and close outgroups *Joinvillea* and *Carex* to look for potential evidence of changes in TSH4 binding (Fig. 5H). We identified two syntenous conserved noncoding sequences (CNSs) in all promoters, with the exception of *Carex*, which lacked one of the CNSs. We also mapped the distribution of the consensus SBP binding sites [GTAC; (*64*)], and found that they were distributed randomly throughout all promoters. In addition, we observed a marked cluster of potential SBP binding sites in some promoters. In maize *tsh1*, this cluster overlapped with the known binding site of TSH4. A similar cluster was identified in all the core grass *NTT* promoters, but reduced (*Pharus* and *Joinvillea*) or lacking (*Carex*) outside the core grasses. While the relevance of the apparent gain in TSH4 binding site in grass *NTT* genes to in vivo binding dynamics will require further confirmation, the pattern we see is consistent with recruitment of *tsh1* by *tsh4* early in the evolution of the grass family.

**Fig. 6.**
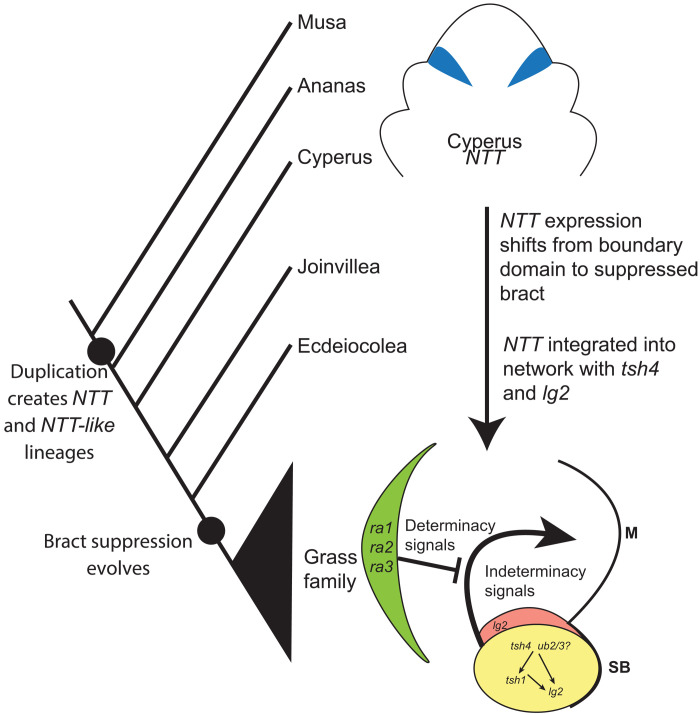
Proposed evolutionary origin and core network of bract suppression in the grass family. SB, suppressed bract; M, meristem.

Together, these results are consistent with a model in which a gene duplication event created the *NTT* lineage, which maintained the ancestral boundary domain function and expression. Later, the *NTT* lineage was recruited, possibly by *tsh4*, to the bract where it maintained its ancestral boundary domain protein function leading to inhibition of bract growth (Fig. 6).

## DISCUSSION

We identified a core network composed of *tsh1*, *tsh4*, and *lg2* that redundantly regulate bract suppression and branch meristem determinacy. This network is hierarchical with *tsh4* upstream of *tsh1*, and both *tsh4* and *tsh1* are upstream of *lg2* (Fig. 6). *tsh1* and orthologous *NTT* genes in the grass family likely neofunctionalized, shifting an ancestral boundary domain gene to the bracts of the grass inflorescence, where they inhibit bract growth and promote branch indeterminacy (Fig. 6). These results provide insights into the regulatory structure of the bract suppression network, its evolutionary origin, and possible roles for bract suppression in grass inflorescence architecture.

### Synergistic interactions of *tsh1*, *tsh4*, and *lg2* suggest redundant roles in a common bract suppression/branch determinacy network

Pairwise double mutant interactions of *tsh1*, *tsh4*, and *lg2* all show significant synergistic interactions that enhance bract growth and inhibit branch growth. While synergistic genetic interactions are common among duplicate transcription factors that act redundantly, *tsh1*, *tsh4*, and *lg2* are each representatives of unrelated transcription factor families. A reasonable explanation for these synergistic interactions among unrelated genes is that they are tightly integrated in a common network required for bract suppression and branch growth. In addition to the synergistic interactions, double mutant populations among these genes repeatedly show dosage effects despite the fact that each of these mutants is recessive on its own. This may indicate that a threshold level of *tsh1/tsh4/lg2* activity is required for normal inflorescence development, and below this level, any further reduction of these genes compromises signaling through their common network and contributes in a dosage-dependent fashion to bract and branching phenotypes. The structure and possible evolution of this network, which appears to be unique to the grasses, is discussed below.

### *tsh4* and *ub2*/*3* are key regulators of bract suppression associated with phase change

The transition from vegetative to reproductive development in grasses involves marked changes to plant development including a change in phyllotaxy, suppression of internode elongation, inhibition of leaf (bract) growth, and associated promotion of branch meristem growth. The reproductive transition is the final in a series of phase changes that are regulated by a developmental mechanism that appears to be largely conserved across the angiosperms. This mechanism involves competing sets of transcription factors, each, in turn, regulated by microRNAs. The adult and floral phases are promoted by miR172 regulation of its AP2 family targets, while the juvenile phase is promoted by miR156 regulation of its SPL targets (*65*). Of the many morphological changes that occur during the transition to flowering, the miR156 target *tsh4* and paralogs *ub2* and *ub3* regulate bract suppression, tassel branching, and meristem size (*7*). Thus, *SPL* genes are largely conserved in their phase transition roles, while their downstream targets can change in accordance with lineage-specific developmental differences involved in floral transition such as bract suppression. Our data suggest that *tsh1* and *lg2* are downstream targets of *tsh4* in maize, and possibly throughout the grass family. Given the residual expression of *tsh1* in *tsh4* mutants and the genetic redundancy of *ub2* and *ub3* with *tsh4* in bract suppression (*7*), *ub2* and *ub3* are strong candidates for promoting *tsh1* expression in parallel with *tsh4*. Our results are consistent with a model in which bract suppression in the grasses came under the control of *tsh4*/*ub2*/*ub3* as part of the reproductive transition placing *tsh4* at the top of a regulatory hierarchy of bract suppression and the associated promotion of inflorescence meristem indeterminacy (Fig. 6). This contrasts with the convergent suppression of bracts in arabidopsis, which is regulated by distinct genetic mechanisms with a large role played by the floral meristem identity gene *LFY*.

### *tsh1*/*NTT* genes in the grass family likely maintain boundary domain functions

Our data suggest that the novel function of *tsh1/NTT* genes in grass bract suppression evolved from an ancestral role in boundary domain promotion. Despite the evolution of a novel expression domain in the suppressed bract, TSH1 appears to have largely maintained the molecular activity of a boundary domain gene. While boundary domain genes include a diverse set of transcription factors, they share some molecular and morphogenetic properties including the ability to inhibit cell division and expansion in organ boundaries, direct growth of adjacent tissues, and regulate hormone homeostasis (*46*, *66*, *67*). Thus, boundary domains simultaneously function as growth repressors and nonautonomous signaling centers, roles that evolved convergently for boundary domains in animals (*68*). Transcript profiling of *tsh1* in maize and ectopic expression of TSH1 in arabidopsis are consistent with a model in which TSH1 maintains ancestral boundary domain activities, but transferred them to a lateral organ primordium (bract) suppressing its growth and simultaneously promoting growth in the adjacent branch meristem.

### Developmental regulation of ligules, bracts, and branch meristem determinacy is tightly correlated in the grasses

Previous work in maize has shown a recurring pleiotropy in which ligule mutants also have tassel branching defects (*48*, *49*). Here, we show that, at least for *lg2*, this pleiotropy extends to bract suppression as *lg2* interacts with both *tsh1* and *tsh4* to regulate both branch meristem determinacy and bract suppression. That *tsh1* expression is localized to the ligular boundary domain in both maize and rice (*51*, *52*) is notable in light of the *tsh1 lg2* interaction in inflorescence development, although no consistent functional role for *tsh1* in ligule or auricle development has yet emerged (however, see fig. S7C). It is not immediately clear why bract suppression and inflorescence branching would be under the control of the same genes that are necessary for ligule establishment. Insofar as the ligule can be understood as a boundary that separates the sheath and blade in grass leaves, the correlation of ligule development with bract growth and inflorescence branching further underscores the important role of boundary domain genes in these distinct developmental contexts. While the suppressed bract is a novel trait in the grass family, the origin of ligules is less clear (*69*). Future work exploring the intersection of ligule development and bract suppression in an expanded phylogenetic context may shed light on the molecular and evolutionary mechanisms by which these traits arose and became integrated during grass inflorescence branching.

### Bract suppression may be an indirect effect of creating a branch meristem indeterminacy–promoting region acting in opposition to *ramosa* genes

Inflorescence architecture is highly diverse across the angiosperms, and particularly in the grass family. Establishing this architecture requires regulation of meristem determinacy during ontogeny of the inflorescence. After initiation, lateral meristems can either continue growing and branching (indeterminacy) or form a limited number of floral primordia before the meristem is consumed (determinacy) (*26*).

The size and activity of the meristem is coordinated by complex interactions of nonautonomous factors that signal from multiple domains within and around the meristem. Meristem growth versus non-growth is not a simple switch, but a complex readout of competing signals that either promote or inhibit meristem size (*70*), including signals originating outside the meristem proper from adjacent lateral organs (*71*, *72*). While many aspects of the meristem growth network are likely common to all meristems in the plant, how these meristem dynamics are regulated in different developmental contexts to alter plant architecture is less clear. Maize inflorescence architecture mutants suggest that determinacy of branch meristems in the inflorescence involves additional regions of nonautonomous signaling specific to the grass family.

The *ramosa* (*ra*) genes, *ra1*, *ra2*, and *ra3*, are core regulators of branch meristem determinacy in maize. However, *ra* genes are not expressed in the meristem itself, but rather in a boundary domain adaxial to the meristem (*73*–*75*), suggesting that *ra* genes establish a determinacy signaling center adjacent to the meristem. Our work demonstrates that *tsh1*, *tsh4*, and *lg2* interact in a network necessary not only for bract suppression but also for meristem indeterminacy, thus functioning in opposition to *ramosa* genes. Supporting this, *tsh4* is epistatic to *ra* mutants with respect to inflorescence branch determinacy (*5*). Similarly, the *ra1* branch growth phenotype at the base of the tassel requires *lg2* (*73*). Thus, genetic evidence suggests that the indeterminacy-promoting effects of *tsh4* are negatively regulated by *ra* signaling. Furthermore, *tsh1*, *tsh4*, and *lg2* are not expressed directly in the meristem but act from an adjacent domain, analogous to *ra* genes but in the meristem subtending bract rather than adaxial to the meristem. Inflorescence meristem branching in maize thus appears to be under the control of antagonistic signaling centers loosely analogous to the interaction of signaling domains that internally regulate meristem size (*26*, *76*).

*Ra* mutants have increased branching throughout the inflorescence, while *tsh1*, *tsh4*, and *lg2* indeterminacy defects are largely confined to the basal long branch zone and reduced in the central spike. This suggests that a proximo-distal gradient or threshold along the inflorescence axis affects the meristem determinacy activity of *tsh1*, *tsh4*, and *lg2*. Since *tsh1*, *tsh4*, and *lg2* are expressed in the central spike, it is possible that other factors redundantly promote meristem determinacy in the central spike. Alternatively, the balance of *ra* and *tsh/lg2* signaling could change in response to other factors along the proximo-distal axis. A more careful examination of *tsh* and *ra* genetic interactions could shed light on these dynamics.

While the evidence presented here supports a signaling role for *tsh* genes in the promotion of branch indeterminacy, the nature of the nonautonomous signal originating in the bract is still uncertain. Mobile biomolecules including proteins, small peptides, RNA, hormones, or other small molecules are all possible. An intriguing possibility is that the extensive diversification of branching architecture in the grasses was facilitated by the integration of indeterminacy and determinacy signals emanating from the suppressed bract and *ramosa* genes, respectively, to create a grass-specific mechanism to regulate inflorescence branching. Future work to elucidate the maize bract indeterminacy signal and its interaction with antagonistic determinacy signals will provide a framework for understanding the developmental constraints regulating inflorescence architecture in this agronomically important species.

## MATERIALS AND METHODS

### Plant materials

Two new alleles of *tsh4* were isolated from distinct sources. *tsh4-rm* (originally *tsh2*) was identified in a *Mutator*-transposon active population and was introgressed over five times to the reference B73 background before all experiments described here. In a screen for genetic modifiers of *tsh1-2* (A619 background), a phenotypically weak allele, we identified several *enhancers of tasselsheath1* (*ent*) mutants including one we designated ent*-355, which was subsequently renamed tsh4-ent*-355 based on mapping and allelism with *tsh4-ref*. *lg2-R* was introgressed four times into B73 before generating *lg2; tsh1*-*ref* and *lg2; tsh4-rm* double mutant populations. B73, *tsh1-ref*, *tsh4-rm*, and *tsh1-ref tsh4-rm* mutants used for LM RNA-seq assays were grown in 5-gallon pots in a greenhouse at 24°C with supplemental lights for 16-hour light/8-hour dark period.

### Generating double mutants, genotyping, and phenotypic analysis

*tsh1*-*ref* (B73) was crossed as a female to *tsh4*-*rm* (B73) to generate a *tsh1*/*+*, *tsh4*/*+* segregating population. *lg2*-*R* (B73) was crossed as a female to *tsh1*-*ref* (B73) and *tsh4*-*rm* (B73) to generate *tsh1*/*+*, *lg2*/*+* and *tsh4*/*+*, *lg2*/*+* segregating populations, respectively. Each segregating population was grown at an irrigated field in Spanish Fork, Utah and genotyped by PCR using NEB OneTaq DNA polymerase (see table S13 for primer sequences and genotyping instructions). Mature tassels from individual plants were collected, and tassel-related phenotypes were inspected manually.

### Scanning electron microscopy

Dissected ear and tassel primordia were fixed overnight in FAA (4% formalin, 50% ethanol, 5% acetic acid, and 0.01% Triton X-100), dehydrated through an ethanol series, and transitioned to 100% acetone before drying using a 931.GL Supercritical Autosamdri critical point dryer (Tousimis, Maryland) according to the manufacturer’s protocol. Dried samples were mounted on stubs and sputter-coated with gold:palladium (80:20) with Quorum Q150TES (Quorum Technologies, East Sussex, England), before imaging on a XL30 FEI scanning electron microscope (TSS Microscopy, Hillsboro, OR, USA) with an acceleration voltage of 5 to 30 kV under high vacuum mode (<20 mbar) with a working distance of 10 to 20 mm.

### Laser microdissection and RNA-seq library preparation

LM-seq was performed largely as described in (*77*). Briefly, ear primordia were fixed overnight in 3.5% paraformaldehyde, dehydrated through an ethanol series, and transferred to Histoclear (National Diagnostics) before embedding in paraffin. Embedded samples were sectioned at 5 μm and mounted onto charged HistoBond slides (VWR International). The slides were then subjected to laser microdissection by using a PALM microbeam system (Zeiss). Three biological replicates were prepared per genotype (B73, *tsh1*, *tsh4*, and *tsh1 tsh4*). Approximately 750 μm of cells was harvested for each biological replicate. RNA was extracted from microdissected tissues with the PicoPure RNA Isolation Kit (Life Technologies, Carlsbad, CA) and in vitro amplified using TargetAmp 2-round aRNA Amplification Kit 2.0 (Epicentre, Madison, WI). RNA-seq libraries were constructed using the TruSeq Stranded mRNA Library Prep Kit and quantified on an Agilent bioanalyzer (Agilent), and single-end 125–base pair (bp) sequences were generated on Illumina HiSeq 2500.

### Differential expression analysis of RNA-seq data

Differential expression analysis was performed as previously described (*78*) with minor modifications. Twelve RNA-seq libraries were sequenced, and a total of ~958 million single-end raw reads were obtained with an average of 79.9 million reads per library. The overall quality of our sequencing data was assessed using FastQC, and the raw reads were filtered using Trimmomatic v.0.36 to trim and remove low-quality reads and adapter sequences. The filtered reads were mapped to the maize B73 reference genome version 3, release 31 (AGPv3.31) using STAR aligner v.2.6.0a with default parameter settings. Total mapped and uniquely mapped reads are summarized in table S14. A read count matrix including all samples was generated by aggregating the raw counts of mapped reads for a given gene in each sample using featureCounts with reference to 39,479 maize gene models in AGPv3.31. The read count matrix was subjected to differential gene expression analysis using Bioconductor R package edgeR v.3.22.5. Briefly, genes with ubiquitously low expression were filtered out from the read count matrix to improve differential expressed gene detection sensitivity, and only the genes that had count-per-million value of >0.25 in at least three libraries were retained. This resulted in a filtered read count matrix containing 20,168 expressed genes in the samples (table S1). The filtered read count matrix was normalized for compositional bias between libraries using a trimmed means of *M* values (TMM) method and then used to detect genes with differential expression between pairwise samples. Genes with an adjusted *P* value (*q* value) of ≤0.05 and an absolute value of log_2_ fold changes of ≥1 were considered as differentially expressed.

### Gene coexpression cluster analysis

Coexpression analysis was performed as previously described (*78*) with minor modifications. The 1389 genes that were differentially expressed between the wild type (B73) and the three *tsh* mutants (*tsh1*, *tsh4*, and *tsh1*; *tsh4*) were subjected to coexpression cluster analysis across all samples using Bioconductor R package coseq v1.5.2. The raw read count matrix of the 1389 genes in the 12 RNA-seq libraries was converted into an RPKM (reads per kilobase of transcript per million mapped reads) matrix (table S15) that was then used as an input in coseq for coexpression analysis. Briefly, log centered log ratio (log CLR) transformation and TMM normalization were applied to the gene expression matrix to normalize the expression of genes, and the *K*-means algorithm was used to identify the coexpressed clusters across all samples. A range of clusters from 2 to 20 was tested to identify the optimal number of clusters. The *K*-means algorithm embedded in the coseq() function was repeated for 40 iterations (counts, *K* = 2:20; transformation = “logclr”; norm = “TMM”; model = “kmeans”), and the resulting number of clusters in each run was recorded. The most frequently occurring number of clusters was selected as the optimal number of clusters, and genes that were assigned to these clusters were retained for cluster visualization and GO enrichment analysis.

### GO enrichment analysis

Statistically enriched (overrepresented with an adjusted *P* value of ≤0.05) GO terms for genes differentially expressed between pairwise samples or for genes assigned to certain coexpression clusters were identified using singular enrichment analysis in AgriGO v2.0 at http://systemsbiology.cpolar.cn/agriGOv2/ with default parameter settings. After collapsing and removing redundant or very high-level terms, the most statistically enriched GO terms were plotted in ggplot2 for visualization.

### TSH4 ChIP-PCR

Maize B73 plants were grown in the experimental field of the Plant Gene Expression Center, University of California Berkeley. Young ear primordia smaller than 5 mm were carefully dissected. About 1 g of tissue per biological replicate was fixed in 1% formaldehyde solution for 10 min under vacuum and quenched by adding glycine to a final concentration of 0.1 M. Nuclei extraction and ChIP using the TSH4 antibody were performed as described previously (*78*). Normal goat anti-rabbit IgG was used as a negative control. To validate the putative TSH4-binding targets, three biological replicates of immunoprecipitated DNA in ChIP were applied for each qPCR using respective primer pairs listed in table S13 with Fast Evagreen qPCR mix. Relative enrichment was calculated using the Δ*C*_t_ (threshold cycle) method, and significant difference was evaluated through *t* test between anti-TSH4 ChIPed samples and IgG control.

### LG2 antibody generation and immunolocalization

Full-length LG2 coding sequence was cloned into gateway vector pDEST17. N-terminal HIS-tagged full-length LG2 was expressed in rosetta cells and purified in 8 M urea. The antibodies were produced in guinea pigs (Cocalico Biologicals). Whole serum was tested for reactivity via dot blot and then purified first against HIS protein and then against the N terminus of LG2 (residues: 1 to 200, cloned into pDEST15, produced in rosetta cells) fused to glutathione *S*-transferase (GST) as described in (*5*). Specificity was tested using immunolocalization and Western blot in wild-type and *lg2* tissue. Immunolocalization used a protocol based on (*79*) and was as follows. Slides were deparaffinized using Histoclear and then rehydrated through an ethanol series to water. Samples were then boiled in 10 mM sodium citrate (pH 6) for 10 min to retrieve the antigens. Blocking was carried out in 1% bovine serum albumin (BSA)/phosphate-buffered saline (PBS)/0.3% Triton X-100 for 3 hours. Slides were incubated overnight in the primary antibody, before washing in PBS/0.3% Triton X-100. They were then incubated with secondary antibody (goat anti–guinea pig alkaline phosphatase conjugate; Bethyl, #A60-110AP) at room temperature for 2 hours. Slides were then were incubated in a 1:50 dilution of 5-bromo-4-chloro-3-indolyl phosphate/nitro blue tetrazolium (BCIP/NBT) (Roche, #11681451001) in 0.05 M MgCl_2_/tris-buffered saline (pH 9.5) until a dark precipitate was observed. These slides were then imaged on a Leica MZ16-F dissecting microscope with an attached canon EOS 250D camera in water. The LG2 antibody was used at a 1:300 dilution, and goat anti–guinea pig alkaline phosphatase was used at a 1:400 dilution in 1% BSA in PBS.

### Isolation of *NTT* orthologs from the Poales and phylogenetic analysis

Genomic DNA and/or total RNA were isolated from young inflorescences of *Hyparrhenia hirta*, *Streptochaeta angustifolia*, *Pharus latifolia*, *Joinvillea ascendens*, *Elegia tectorum*, *Georgeantha hexandra*, and *Cyperus papyrus.* cDNA was generated using the SuperScript III First-Strand Kit (Thermo Fisher Scientific) according to the manufacturer’s protocol with a modified polyT (polythymine) primer (table S13). A series of degenerate primers (table S13) designed for the HAN domain or the GATA domain were used in combination with a polyT primer to isolate the 3′ sequence using 3′ RACE (rapid amplification of cDNA ends), while primers designed to CNSs in the 5′ promoter with gene-specific reverse primers were used to isolate the 5′ end of genes where possible. These amplified sequences were aligned using CLUSTALX, and the resulting alignment was adjusted by hand using MacClade to create a preliminary alignment. This preliminary alignment was used as the prior to search the Gramene and Phytozome coding sequence databases using a hidden Markov model in HMMER v.3.1b2 to isolate *NTT/HAN* orthologs from *A. thaliana*, *Populus trichocarpa*, *Selaginella moellendorffii*, *Physcomitrella patens*, *M. polymorpha*, *Amborella trichopoda*, *Zea mays*, *Miscanthus sinensis*, *Sorghum bicolor*, *Setaria italica*, *Panicum virgatum*, *Brachypodium distachyon*, *Hordeum vulgare*, *Triticum aestivum*, *Oryza sativa*, *Musa acuminata*, *Dioscorea rotunda*, and *Ananas comosus*.

All identified *NTT/HAN* orthologs were aligned using MAFFT v.7.313, which was then filtered for homoplastic positions by Noisy v.1.5.12. Last, the alignment was tested for the best substitution model and used to infer a maximum-likelihood gene tree and 1000 bootstrap replicates using IQTree v.1.6.3. The best-fit model selected by IQTree was TVM+F+R3. The tree was visualized using R v.4.0.2., and a subclade containing the gene of interest (maize TSH1) out to the nearest outgroup clade (containing genes from *S. moellendorffii*, *P. patens*, and *M. polymorpha*) was selected for further refinement. The process described above was repeated but using only the genes included in the subclade as an input to MAFFT. Last, *tsh1* orthologous sequences identified from the recently published *Cyperus littledalei* genome (*80*) were manually aligned to the final alignment using Mesquite v.3.61, and this alignment was used to create the tree using IQTree as described above.

### In situ hybridization

An antisense T7 probe, labeled with dig-UTP (Roche), was synthesized for the full-length cDNA for *CyperusNTT* using the Invitrogen SuperScript III Kit according to the manufacturer’s protocol. Tissue preparation and in situ hybridization were performed as previously described (*3*).

### Transgenic Arabidopsis

Coding sequence for *tsh1* and *HAN* was amplified from cDNA using primers (table S13) with 5′ Xho I (5′) and 3′ Bam HI (3′) sites and cloned into the pBJ36 vector downstream of the 10-OP promoter. Not I fragments containing the 10-OP:*tsh1* and 10-OP:*HAN* gene promoter fusions were then subcloned from pBJ36 into the pMLBART27 binary vector and subsequently transformed into *Agrobacterium* and used to transform wild-type *A. thaliana* Col. Transgenic lines containing 10-OP:*tsh1* and 10-OP:*HAN* were crossed to the pAP3:Lhg4 driver line (*71*).

### Promoter analysis

Genomic sequence containing 5 kb of 5′ promoter region upstream of the start codon for *tsh1* (*Z. mays*), sorghum *NTT* (*S. bicolor*), setaria *NTT* (*S. italica*), *NL1* (*O. sativa*), brachypodium *NTT* (*B. distachyon*), *P. latifolia NTT*, *J. ascendens NTT*, and *Carex littledalei NTT* isolated the respective published genomes of each species. Pairwise alignments of all promoters were performed with blastn to identify significant stretches of nucleotide identity spanning at least 15 bp. After aligning all pairs of sequences, two regions of similarity from blastn alignments were identified, with only *Carex* lacking one of these. Since they were syntenously arranged in all promoters, we designated these region CNSs. CNS alignments and potential binding sites are provided in fig. S10.

## Supplementary Materials

### This PDF file includes:

Figs. S1 to S10 References

## Notes

View/request a protocol for this paper from *Bio-protocol*.
