# Supplementary material for: Boundary domain genes were recruited to suppress bract growth and promote branching in maize
Source: Sci Adv. 2022 Jun 15;8(24):eabm6835. doi: 10.1126/sciadv.abm6835 (PMC9200273; doi:10.1126/sciadv.abm6835)
Supplement: Supplementary file 1 — Figs. S1 to S10 References [file sciadv.abm6835_sm.pdf]

Science Advances
AAAS

Supplementary Materials for

Boundary domain genes were recruited to suppress bract growth and promote branching in maize

Yuguo Xiao *et al.*

Corresponding author: Clinton Whipple, whipple@byu.edu

Sci. Adv. **8**, eabm6835 (2022)
DOI: 10.1126/sciadv.abm6835

The PDF file includes:

Figs. S1 to S10
Legends for tables S1 to S16
References

Other Supplementary Material for this manuscript includes the following:

Tables S1 to S16

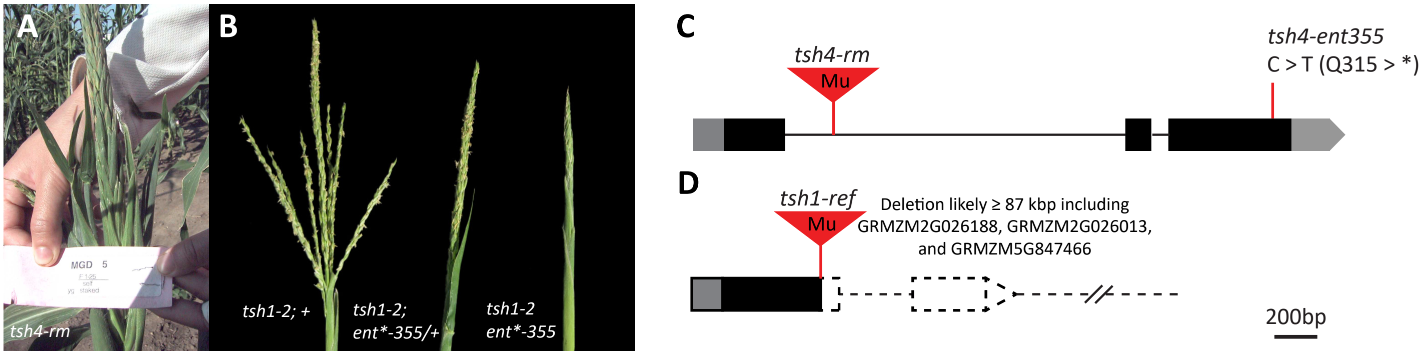

Fig. S1. New alleles of *tsh1* and *tsh4*. (**A**) Tassel phenotype of *tsh4-rm* (originally designated *tsh2-1*) in an undetermined *Mu* active background. (**B**) Tassel of weak *tsh1-2* allele (A619), left, used as the genetic background for screen for *tsh1* modifiers. The phenotype is limited to occasional bracts and slight reduction in branching. Right, increased bract formation and loss of branching in the homozygous *tsh1-2 ; enhancer of tsh1*-355* (*ent*-355*) double mutant. *ent*-355* is allelic to *tsh4* and was redesignated *tsh4-ent355*. Center, reduced branching phenotype of *tsh1-2* homozygote also heterozygous for *tsh4-ent355*/+. (**C**) Lesions identified in new *tsh4* alleles *tsh4-rm* and *tsh4-ent355*. (**D**) Mutator insertion followed by large deletion identified in previously uncharacterized *tsh1-ref* allele.

In a previous screen for maize bract suppression mutants (*3*), we described five *tsh* loci (*tsh1-tsh5*), of which *tsh1* and *tsh4* have been cloned (*3, 5*). We focused next on the *tsh2-1* mutant, which was isolated in a screen of *Mutator* transposon active families (*81*). Our original attempts to map *tsh2* showed no linkage with *tsh1* or *tsh4* and complementation of *tsh3* and *tsh5* (Whipple et al., 2010). In contrast with these results, a subsequent bulked-segregant analysis followed by fine-mapping placed *tsh2* on chromosome 7 between SSR markers *bnlg434* and *umc1408*, a 10 Mbp region that contains *tsh4*. Sequencing of *tsh4* in our *tsh2-1* allele revealed a *Mutator* transposon in the first intron (**A** and **C**). F1 progeny of a cross with *tsh4-Ds* (*5*) confirmed that these alleles fail to complement, further supporting that *tsh2-1* is allelic with *tsh4*. Consequently we renamed *tsh2-1* as *tsh4-rm*.

In an EMS mutagenesis screen for modifiers of *tsh1* we identified a strong enhancer provisionally named *enhancer of tsh1*-355* (*ent*-355*) (**B**). This enhancer segregated as a semi-dominant factor in the homozygous *tsh1-2* (A619) background. *tsh1-2/tsh1-2*; *ent*-355*/+ plants showed a marked reduction in branching relative to *tsh1-2* alone, and double mutant *tsh1-2* ; *ent*-355* plants had no branches and many more de-repressed bracts. Bulked-segregant analysis placed *ent*-355* on chromosome 7 near *tsh4*, and a cross with *tsh4* failed to complement, indicating that these mutants are also allelic. Sequencing revealed a C>T transition resulting in an early stop codon of *tsh4*, so we designated this mutant *tsh4-ent*355* (**C**).

The transposon insertion and associated deletion causing *tsh1-ref* was inferred by PCR amplification with MuTIR6 primers and *tsh1* specific 5' primers, as well as the inability to amplify *tsh1* sequence by PCR 3' of the *Mu* insertion. The extent of the deletion was determined by examining expression of neighboring genes from our *tsh1-ref* bract LM RNA-seq data-set showing three genes 3' to *tsh1* with no transcripts in *tsh1-ref* despite detectable expression in B73 (see table S16).

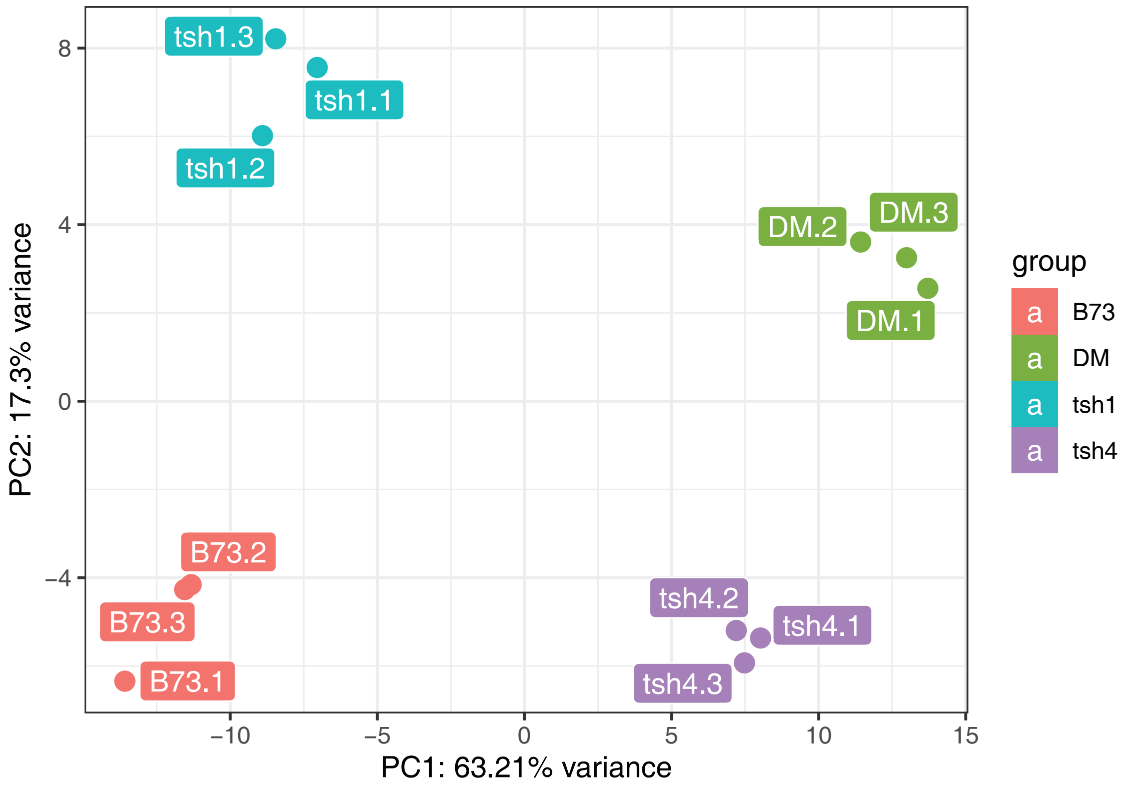

Fig. S2. Principal component analysis of 12 RNAseq libraries made from laser-microdissected bract primordium of B73, *tsh1*, *tsh4* and *tsh1 tsh4* double mutant (DM). Principal components (PC) 1 and PC2 are graphically visualized. Colored dots represent individual biological replicates. Each biological replicate was specified with the corresponding sample name followed by a dot symbol and a unique numeric number.

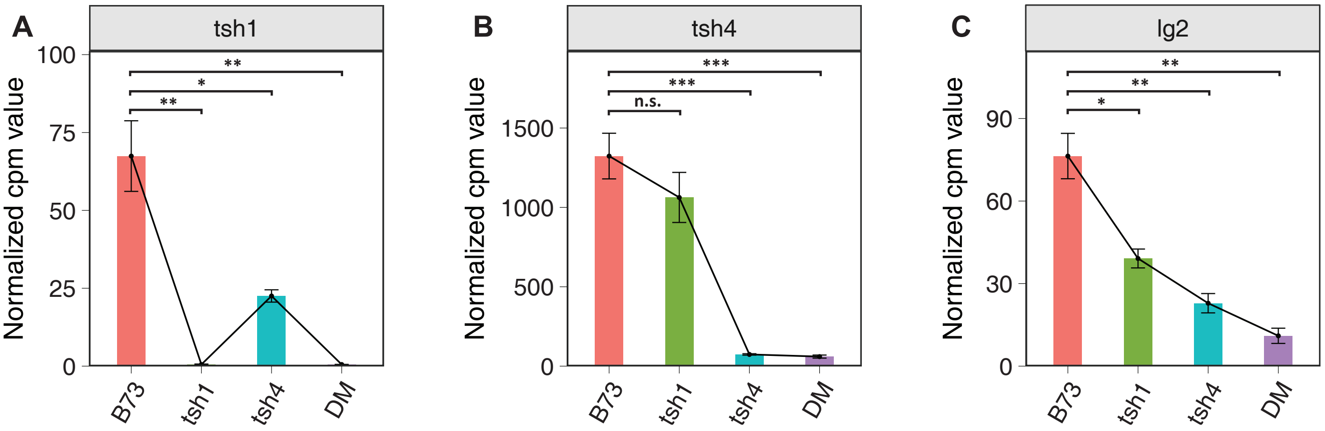

Fig. S3. Expression of *tsh1*, *tsh4* and *lg2* in bracts of B73 and *tsh* mutants. (**A-C**) Expression of *tsh1* (A), *tsh4* (B) and *lg2* (C) in bracts of B73 and *tsh* mutants. Samples are bract primordium of B73, *tsh1*, *tsh4* and *tsh1 tsh4* double mutant (DM). Data are normalized CPM (count-per-million) values based on edgeR output. Data are means ± standard error of three biological replicates. n.s., not significant, *$p < 0.05$, **$p < 0.01$, ***$p < 0.001$; two-tailed Student's t-test.

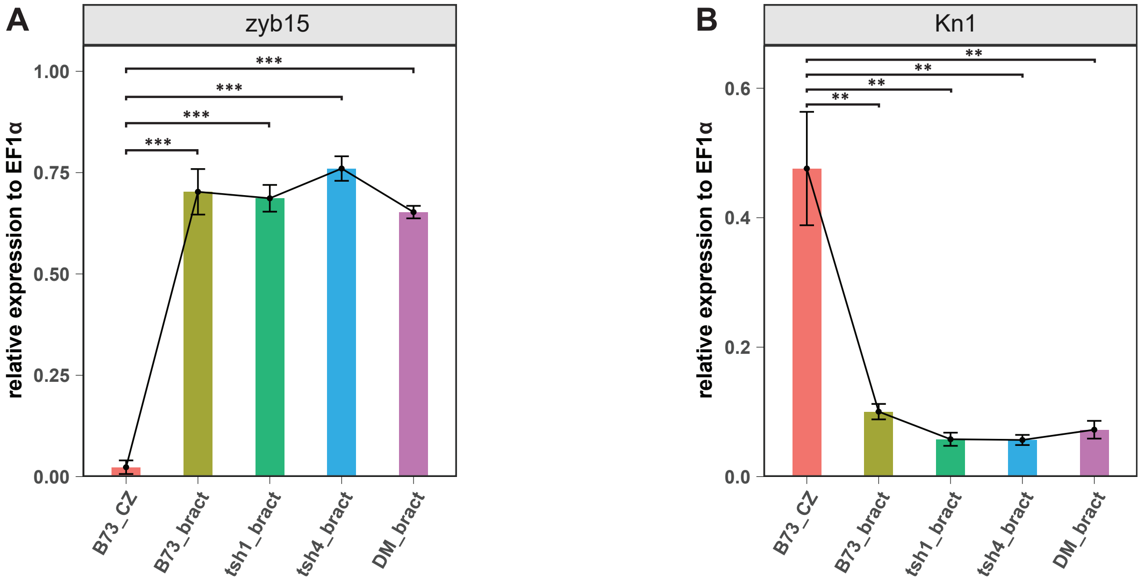

Fig S4. Expression value of *zyb15* and *Kn1* in maize vegetative shoot apex and bracts. (A-B) Relative expression value of *zyb15* (A) and *Kn1* (B) relative to a housekeeping gene *EF1α* in maize vegetative shoot apex and bracts. B73_CZ, center zone of B73 shoot apex; B73_bract, B73 bract; tsh1_bract, *tsh1* bract; tsh4_bract, *tsh4* bract; DM_bract, bract of *tsh1*; *tsh4* double mutant. The expression value of *zyb15*, *Kn1* and *EF1α* in the center zone of B73 vegetative shoot apex was retrieved from Knauer et.al (*82*). EF1α, elongation factor 1-alpha. Data are means ± standard error of three biological replicates. **p<0.01, ***p< 0.001; two-tailed Student's t-test.

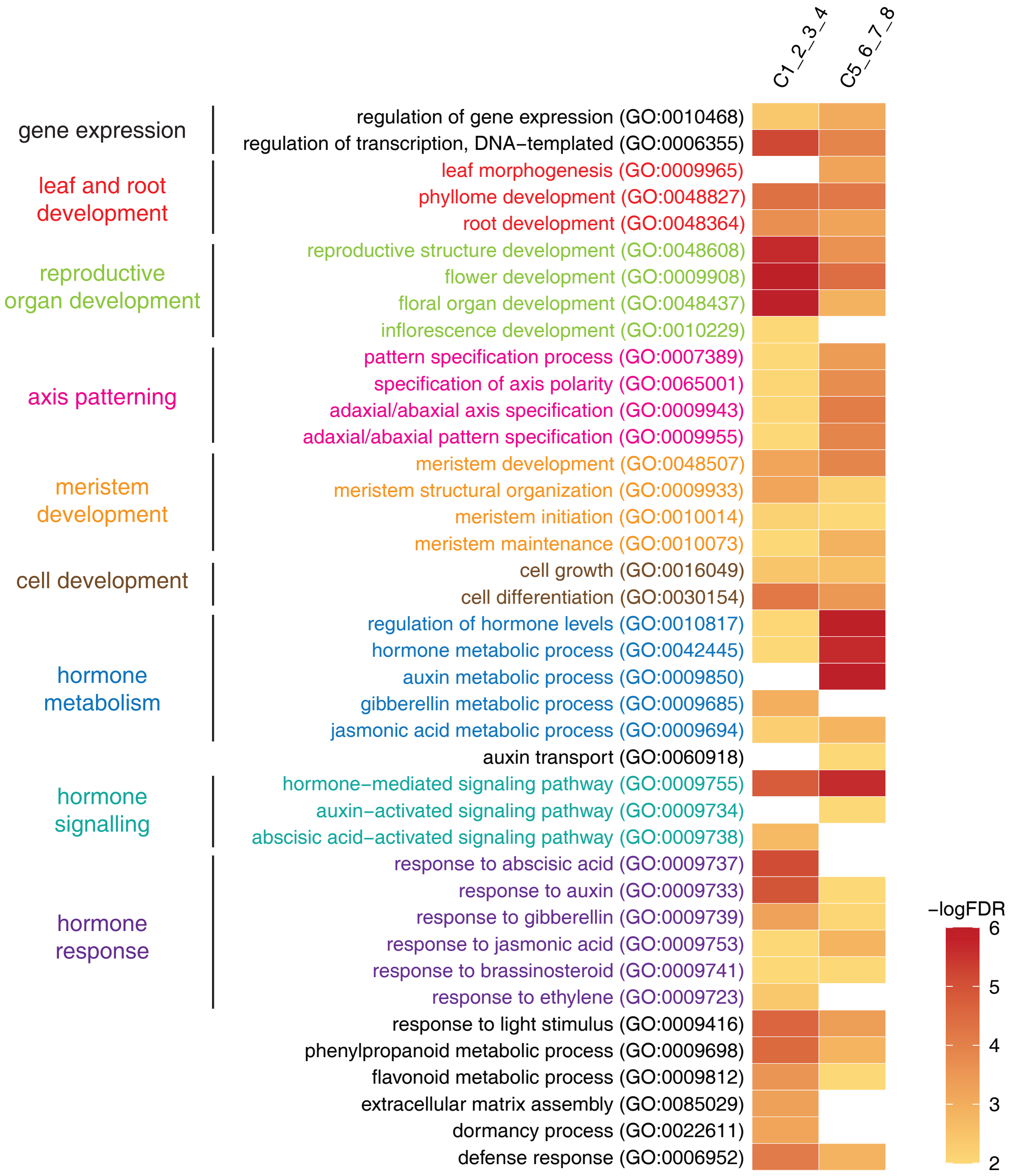

Fig. S5. Over-represented gene ontology terms in the co-expression clusters in Fig. 2F. Gene ontology (GO) enrichment analysis was performed with genes from clusters 1-4 (C1_2_3_4) and 5-8 (C5_6_7_8) in Figure 2F. Statistically enriched GO terms were identified using Singular Enrichment Analysis in AgriGO v2.0 at http://systemsbiology.cau.edu.cn/agriGOv2/ with default parameter settings. GO terms with false discovery rate (FDR) ≤ 0.01 (−logFDR ≥ 2) were considered as significantly enriched. After collapsing and removing redundant or very high-level terms, the most statistically enriched GO terms were plotted.

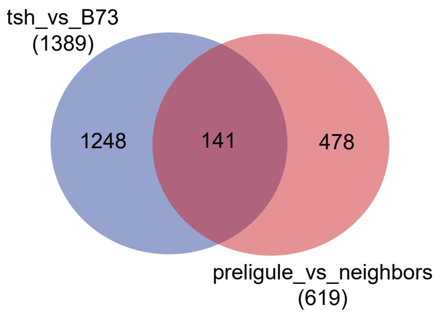

Fig.S6. Venn diagrams showing common and uniquely differentially expressed genes in *tsh* mutants, *lg1* mutant and the preligule region of wildtype. tsh_vs_B73 stands for the 1389 genes differentially expressed in the bracts of *tsh* mutants (*tsh1*, *tsh4* and *tsh1 tsh4* double mutants) compared to that in the wildtype (B73). preligule_vs_neighbors represents the 619 genes differentially expressed in the preligule region compared to that in the neighboring regions (blade and sheath) of wildtype maize developing leaf. lg1_vs_wt represents the 96 genes differentially expressed in the leaf primordium of *lg1* mutant compared to that in the wildtype. Genes differentially expressed in the preligule region or in the *lg1* were retrieved from Johnston et al. (*52*).

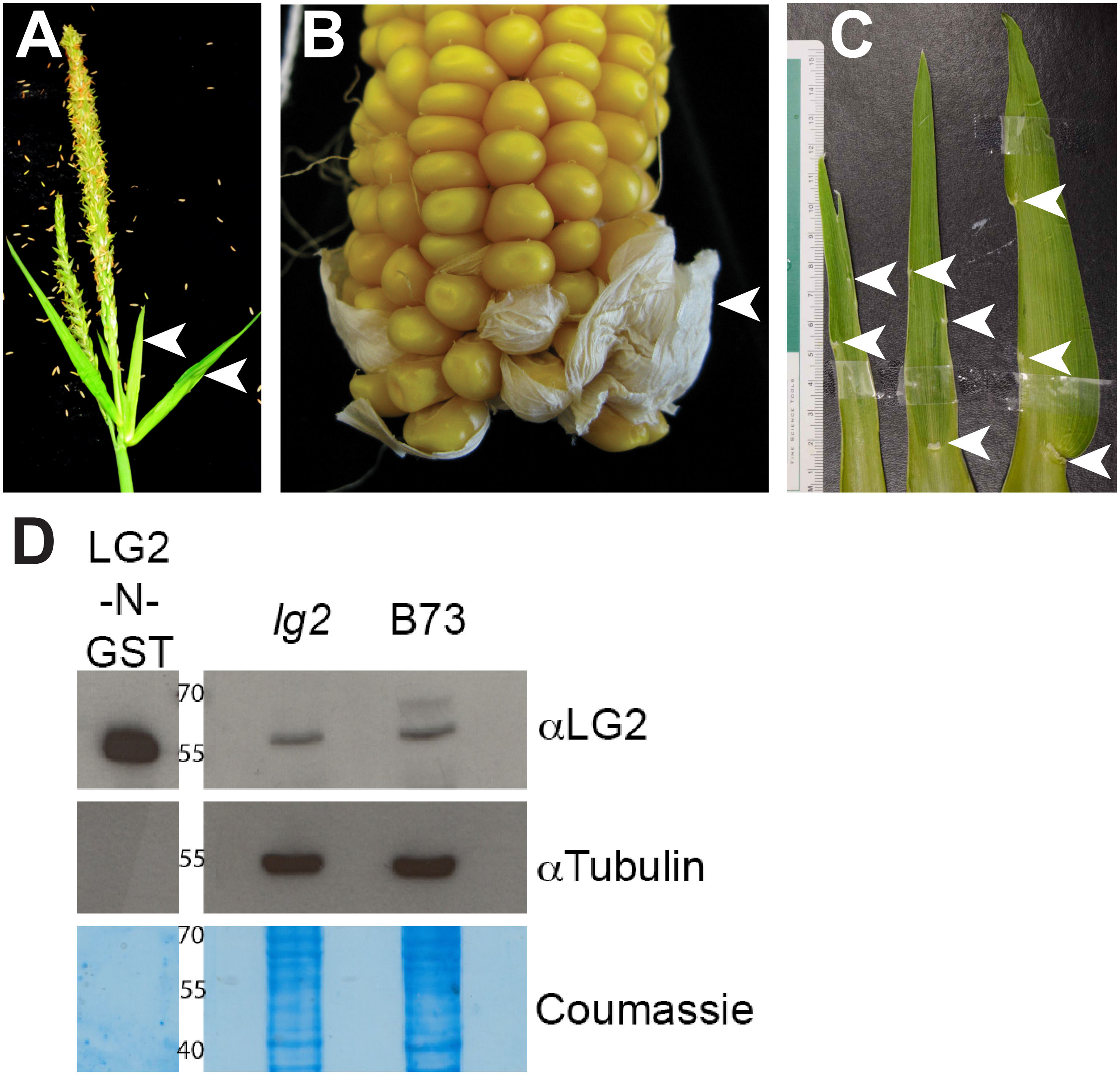

Fig. S7. *lg2* bract phenotype, *tsh1* ligule phenotype and specificity of LG2 antibody. (**A**) Tassel and (**B**) ear inflorescence phenotype of occasional *lg2* mutant showing ectopic bract growth (arrowheads). (**C**) Displaced ligule/auricle tissue of flag leaves of *tsh1-2* mutants. (**D**) Western blot of crude protein extract from *lg2* (reference allele, which is not a protein null allele) and B73 2-week-old shoot apices. Each sample represents protein extract from a pool of 4 individuals. The N terminus of LG2 fused to GST (LG-N-GST) was used to produce the antibody and was used as a positive control in the western blot. The LG2 protein has a predicated size of 57KDa, the upper band in the B73 sample suggests that LG2 is post-translationally modified. Anti-TUBULIN was used as a protein load control. All images are of blots taken from the same gel.

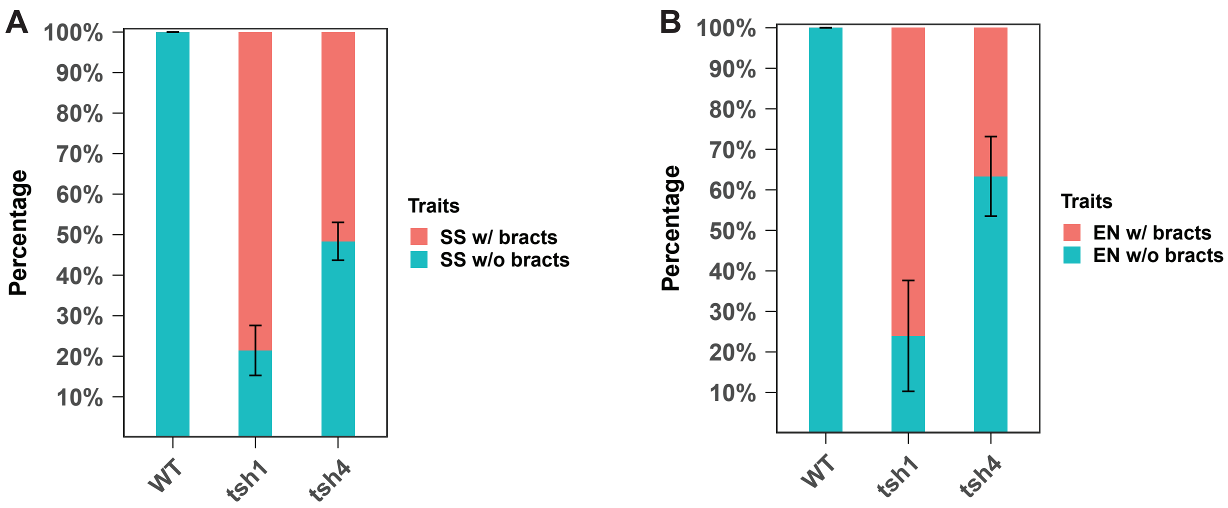

Fig. S8. Percentage of empty nodes and solitary spikelets with or without subtending bracts in wildtype, *tsh1* and *tsh4* mutants. (**A-B**) Percentage of solitary spikelets (A) and empty nodes (B) with or without subtending bracts. Six to eight individual plants per genotype were manually inspected. WT, wildtype. SS w/ bracts, solitary spikelets with subtending bracts; SS w/o bracts, solitary spikelets without subtending bracts; EN w/ bracts, empty nodes with subtending bracts; EN w/o bracts, empty nodes without subtending bracts.

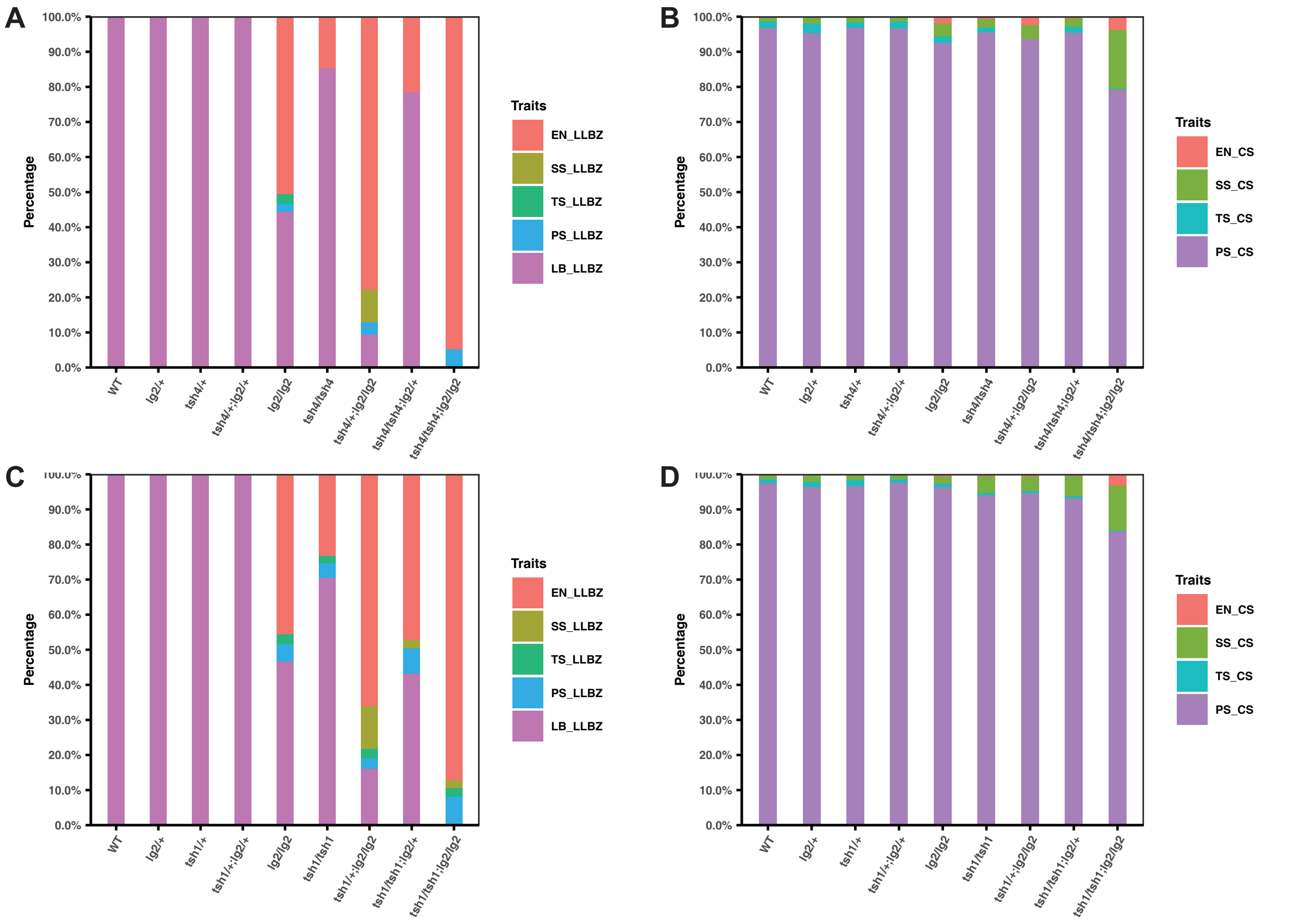

Fig. S9. Proximo-distal determinacy defects in *tsh4 lg2* and *tsh1 lg2* segregating populations. (**A** to **D**) Percentage of long tassel branch, paired spikelet, triple spikelet, solitary spikelet and empty node in the long lateral branch zone (A and C) and central spike above the long lateral branch zone (B and D) in *tsh4 lg2* (A and B) and *tsh1 lg2* (C and D) segregating populations. EN, empty node; SS, solitary spikelet; TS, triple spikelet; PS, paired spikelet; LB, long tassel branch; LLBZ, long lateral branch zone; CS, central spike above the long lateral branch zone.

Conserved Non-coding Sequence (CNS) I

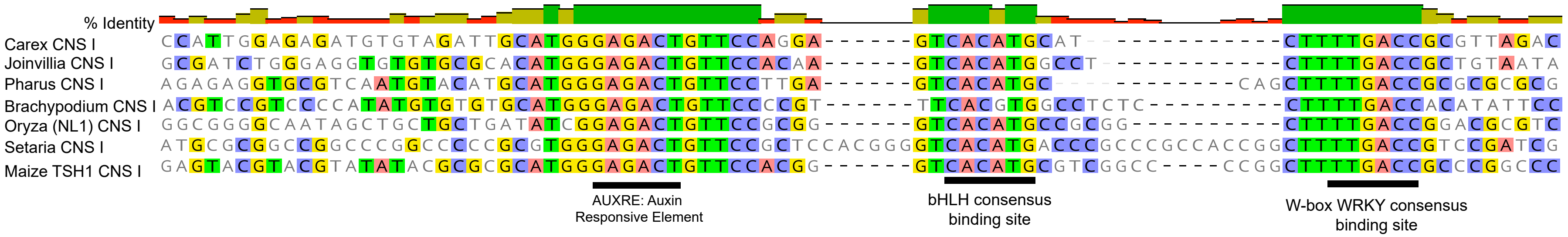

Conserved Non-coding Sequence (CNS) II

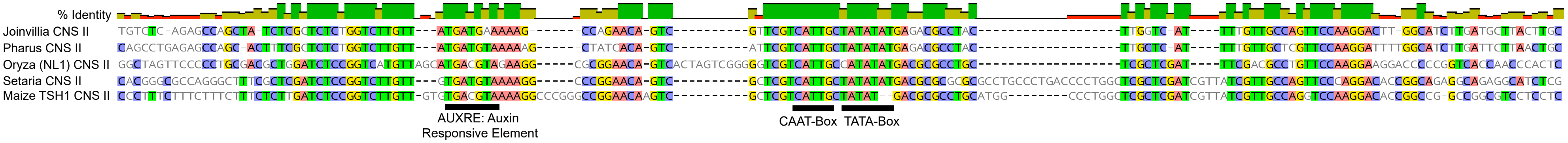

Fig. S10. Conserved non-coding sequences in the promoters of *NTT* orthologs in the grasses and close outgroups. Alignments of Conserved Non-coding Seqences (CNSs) as indicated in Fig. 5H. Potential binding sites of TCP and bHLH transcription factors are indicated for CNS I, while binding sites typical of a core promoter element (CAAT-box and TATA-box) as well as an auxin responsive element are indicated for CNS II. Neither CNS overlapped with consensus SBP binding sites, nor published-DAP-seq peaks.

Supplementary table legends

Table S1. Genes expressed in bracts of B73, *tsh1*, *tsh4* and *tsh1 tsh4* double mutant.

Table S2. Genes differentially expressed between *tsh1* and B73.

Table S3. Genes differentially expressed between *tsh4* and B73.

Table S4. Genes differentially expressed between *tsh1 tsh4* double mutant and B73.

Table S5. Genes differentially expressed between B73 and at least one of the *tsh* mutants.

Table S6a. Number of genes grouped into individual clusters by gene co-expression analysis.

Table S6b. Gene co-expression analysis on genes differentially expressed in at least one *tsh* mutant.

Table S6c. Genes co-expressed in cluster 1, 2, 3 and 4.

Table S6d. Genes co-expressed in cluster 5, 6, 7 and 8.

Table S6e. Genes co-expressed in cluster 9 and 10.

Table S7. Shared differentially expressed genes in preligule region and *tsh* mutants.

Table S8. Shared differentially expressed genes in *lg1* and *tsh* mutants.

Table S9. Phenotypic characterization of *tsh1*/+; *lg2*/+ segregating population.

Table S10. Phenotypic characterization of *tsh4*/+; lg2/+ segregating population.

Table S11. Bract phenotype characterization of *tsh1*/+; *lg2*/+ segregating population.

Table S12. Bract phenotype characterization of *tsh4*/+; *lg2*/+ segregating population.

Table S13. PCR primers used in this study.

Table S14. Summary statistics for RNA-sequencing and mapping.

Table S15. Gene expression matrix on genes differentially expressed between B73 and *tsh* mutants.

Table S16. Expression of *tsh1* neighboring genes in bracts of B73 and *tsh1-ref*.

Other Supplementary Materials for this manuscript include the following:

Table S1 to S16 (a separate .xlsx file)

REFERENCES AND NOTES

1. F. Weberling, *Morphology of Flowers and Inflorescences* (Cambridge Univ. Press, 1989).

2. M. K. Ritter, C. M. Padilla, R. J. Schmidt, The maize mutant barren stalk1 is defective in axillary meristem development. *Am. J. Bot.* **89**, 203–210 (2002).

3. C. J. Whipple, D. H. Hall, S. DeBlasio, F. Taguchi-Shiobara, R. J. Schmidt, D. P. Jackson, A conserved mechanism of bract suppression in the grass family. *Plant Cell* **22**, 565–578 (2010).

4. I. A. Al-Shehbaz, M. A. Beilstein, E. A. Kellogg, Systematics and phylogeny of the Brassicaceae (Cruciferae): An overview. *Plant Syst. Evol.* **259**, 89–120 (2006).

5. G. Chuck, C. Whipple, D. Jackson, S. Hake, The maize SBP-box transcription factor encoded by tasselsheath4 regulates bract development and the establishment of meristem boundaries. *Development* **137**, 1243–1250 (2010).

6. G. Chuck, A. M. Cigan, K. Saeteurn, S. Hake, The heterochronic maize mutant Corngrass1 results from overexpression of a tandem microRNA. *Nat. Genet.* **39**, 544–549 (2007).

7. G. S. Chuck, P. J. Brown, R. Meeley, S. Hake, Maize SBP-box transcription factors unbranched2 and unbranched3 affect yield traits by regulating the rate of lateral primordia initiation. *Proc. Natl. Acad. Sci. U.S.A.* **111**, 18775–18780 (2014).

8. D. Weigel, J. Alvarez, D. R. Smyth, M. F. Yanofsky, E. M. Meyerowitz, *LEAFY* controls floral meristem identity in Arabidopsis. *Cell* **69**, 843–859 (1992).

9. S. R. Hepworth, Y. Zhang, S. McKim, X. Li, G. W. Haughn, BLADE-ON-PETIOLE–dependent signaling controls leaf and floral patterning in Arabidopsis. *Plant Cell* **17**, 1434–1448 (2005).

10. M. Norberg, M. Holmlund, O. Nilsson, The BLADE ON PETIOLE genes act redundantly to control the growth and development of lateral organs. *Development* **132**, 2203–2213 (2005).

11. K. D. Allen, I. M. Sussex, *Falsiflora* and *anantha* control early stages of floral meristem development in tomato (*Lycopersicon esculentum* Mill.). *Planta* **200**, 254–264 (1996).

12. N. Molinero-Rosales, M. Jamilena, S. Zurita, P. Gómez, J. Capel, R. Lozano, FALSIFLORA, the tomato orthologue of FLORICAULA and LEAFY, controls flowering time and floral meristem identity. *Plant J.* **20**, 685–693 (1999).

13. J. Hofer, L. Turner, R. Hellens, M. Ambrose, P. Matthews, A. Michael, N. Ellis, UNIFOLIATA regulates leaf and flower morphogenesis in pea. *Curr. Biol.* **7**, 581–587 (1997).

14. K. Ikeda-Kawakatsu, M. Maekawa, T. Izawa, J.-I. Itoh, Y. Nagato, ABERRANT PANICLE ORGANIZATION 2/RFL, the rice ortholog of Arabidopsis LEAFY, suppresses the transition from inflorescence meristem to floral meristem through interaction with APO1. *Plant J.* **69**, 168–180 (2012).

15. K. Bomblies, R.-L. Wang, B. A. Ambrose, R. J. Schmidt, R. B. Meeley, J. Doebley, Duplicate FLORICAULA/LEAFY homologs zfl1 and zfl2 control inflorescence architecture and flower patterning in maize. *Development* **130**, 2385–2395 (2003).

16. Z. Dong, W. Li, E. Unger-Wallace, J. Yang, E. Vollbrecht, G. Chuck, Ideal crop plant architecture is mediated by *tassels replace upper ears1*, a BTB/POZ ankyrin repeat gene directly targeted by TEOSINTE BRANCHED1. *Proc. Natl. Acad. Sci. U.S.A.* **114**, E8656–E8664 (2017).

17. M. Xu, T. Hu, J. Zhao, M.-Y. Park, K. W. Earley, G. Wu, L. Yang, R. S. Poethig, Developmental functions of miR156-regulated *SQUAMOSA PROMOTER BINDING PROTEIN-LIKE (SPL)* genes in *Arabidopsis thaliana*. *PLOS Genet.* **12**, e1006263 (2016).

18. S. Schwarz, A. V. Grande, N. Bujdoso, H. Saedler, P. Huijser, The microRNA regulated SBP-box genes *SPL9* and *SPL15* control shoot maturation in Arabidopsis. *Plant Mol. Biol.* **67**, 183–195 (2008).

19. T. Nawy, M. Bayer, J. Mravec, J. Friml, K. D. Birnbaum, W. Lukowitz, The GATA factor *HANABA TARANU* is required to position the proembryo boundary in the early *Arabidopsis* embryo. *Dev. Cell* **19**, 103–113 (2010).

20. L. Ding, S. Yan, L. Jiang, W. Zhao, K. Ning, J. Zhao, X. Liu, J. Zhang, Q. Wang, X. Zhang, *HANABA TARANU (HAN)* bridges meristem and organ primordia boundaries through *PINHEAD*,

JAGGED, *BLADE-ON-PETIOLE2* and *CYTOKININ OXIDASE 3* during flower development in *Arabidopsis*. *PLOS Genet.* **11**, e1005479 (2015).

21. X. Zhang, Y. Zhou, L. Ding, Z. Wu, R. Liu, E. M. Meyerowitz, Transcription repressor HANABA TARANU controls flower development by integrating the actions of multiple hormones, floral organ specification genes, and GATA3 family genes in *Arabidopsis*. *Plant Cell* **25**, 83–101 (2013).

22. E. S. Coen, J. M. Nugent, Evolution of flowers and inflorescences. *Development* **1994**, 107–116 (1994).

23. G. Chuck, E. Bortiri, The unique relationship between tsh4 and ra2 in patterning floral phytomers. *Plant Signal. Behav.* **5**, 979–981 (2010).

24. J. W. Chandler, Floral meristem initiation and emergence in plants. *Cell. Mol. Life Sci.* **69**, 3807–3818 (2012).

25. D. A. Baum, C. D. Day, Cryptic bracts exposed: Insights into the regulation of leaf expansion. *Dev. Cell* **6**, 318–319 (2004).

26. C. J. Whipple, Grass inflorescence architecture and evolution: The origin of novel signaling centers. *New Phytol.* **216**, 367–372 (2017).

27. D. Jackson, B. Veit, S. Hake, Expression of maize KNOTTED1 related homeobox genes in the shoot apical meristem predicts patterns of morphogenesis in the vegetative shoot. *Development* **120**, 405–413 (1994).

28. J. Strable, J. G. Wallace, E. Unger-Wallace, S. Briggs, P. J. Bradbury, E. S. Buckler, E. Vollbrecht, Maize *YABBY* genes *drooping leaf1* and *drooping leaf2* regulate plant architecture. *Plant Cell* **29**, 1622–1641 (2017).

29. R. Sarojam, P. G. Sappl, A. Goldshmidt, I. Efroni, S. K. Floyd, Y. Eshed, J. L. Bowman, Differentiating Arabidopsis shoots from leaves by combined YABBY activities. *Plant Cell* **22**, 2113–2130 (2010).

30. J. A. Aguilar-Martínez, N. Sinha, Analysis of the role of Arabidopsis class I TCP genes AtTCP7, AtTCP8, AtTCP22, and AtTCP23 in leaf development. *Front. Plant Sci.* **4**, 406 (2013).

31. J. F. Palatnik, E. Allen, X. Wu, C. Schommer, R. Schwab, J. C. Carrington, D. Weigel, Control of leaf morphogenesis by microRNAs. *Nature* **425**, 257–263 (2003).

32. P. Ballester, M. Navarrete-Gómez, P. Carbonero, L. Oñate-Sánchez, C. Ferrándiz, Leaf expansion in Arabidopsis is controlled by a TCP-NGA regulatory module likely conserved in distantly related species. *Physiol. Plant.* **155**, 21–32 (2015).

33. J. H. Kim, D. Choi, H. Kende, The AtGRF family of putative transcription factors is involved in leaf and cotyledon growth in Arabidopsis. *Plant J.* **36**, 94–104 (2003).

34. H. Nelissen, D. Eeckhout, K. Demuynck, G. Persiau, A. Walton, M. van Bel, M. Vervoort, J. Candaele, J. De Block, S. Aesaert, M. Van Lijsebettens, S. Goormachtig, K. Vandepoele, J. Van Leene, M. Muszynski, K. Gevaert, D. Inzé, G. De Jaeger, Dynamic changes in ANGUSTIFOLIA3 complex composition reveal a growth regulatory mechanism in the Maize leaf. *Plant Cell* **27**, 1605–1619 (2015).

35. A. Gallavotti, Q. Zhao, J. Kyozuka, R. B. Meeley, M. K. Ritter, J. F. Doebley, M. E. Pè, R. J. Schmidt, The role of barren stalk1 in the architecture of maize. *Nature* **432**, 630–635 (2004).

36. A. Gallavotti, S. Malcomber, C. Gaines, S. Stanfield, C. Whipple, E. Kellogg, R. J. Schmidt, BARREN STALK FASTIGIATE1 is an AT-hook protein required for the formation of maize ears. *Plant Cell* **23**, 1756–1771 (2011).

37. M. P. Joseph, C. Papdi, L. Kozma-Bognár, I. Nagy, M. López-Carbonell, G. Rigó, C. Koncz, L. Szabados, The Arabidopsis ZINC FINGER PROTEIN3 interferes with abscisic acid and light signaling in seed germination and plant development. *Plant Physiol.* **165**, 1203–1220 (2014).

38. Y. Hu, X. Han, M. Yang, M. Zhang, J. Pan, D. Yu, The transcription factor INDUCER OF CBF EXPRESSION1 interacts with ABSCISIC ACID INSENSITIVE5 and DELLA proteins to fine-tune abscisic acid signaling during seed germination in Arabidopsis. *Plant Cell* **31**, 1520–1538 (2019).

39. K. Chen, G.-J. Li, R. A. Bressan, C.-P. Song, J.-K. Zhu, Y. Zhao, Abscisic acid dynamics, signaling, and functions in plants. *J. Integr. Plant Biol.* **62**, 25–54 (2020).

40. M. G. Heisler, M. E. Byrne, Progress in understanding the role of auxin in lateral organ development in plants. *Curr. Opin. Plant Biol.* **53**, 73–79 (2020).

41. Y. Hu, Q. Xie, N.-H. Chua, The Arabidopsis auxin-inducible gene ARGOS controls lateral organ size. *Plant Cell* **15**, 1951–1961 (2003).

42. P. Hedden, V. Sponsel, A century of gibberellin research. *J. Plant Growth Regul.* **34**, 740–760 (2015).

43. J. Park, K. T. Nguyen, E. Park, J.-S. Jeon, G. Choi, DELLA proteins and their interacting RING finger proteins repress gibberellin responses by binding to the promoters of a subset of gibberellin-responsive genes in Arabidopsis. *Plant Cell* **25**, 927–943 (2013).

44. S. Gazzarrini, Y. Tsuchiya, S. Lumba, M. Okamoto, P. McCourt, The transcription factor FUSCA3 controls developmental timing in Arabidopsis through the hormones gibberellin and abscisic acid. *Dev. Cell* **7**, 373–385 (2004).

45. Z.-L. Zhang, M. Ogawa, C. M. Fleet, R. Zentella, J. Hu, J.-O. Heo, J. Lim, Y. Kamiya, S. Yamaguchi, T.-p. Sun, Scarecrow-like 3 promotes gibberellin signaling by antagonizing master growth repressor DELLA in Arabidopsis. *Proc. Natl. Acad. Sci. U.S.A.* **108**, 2160–2165 (2011).

46. S. R. Hepworth, V. A. Pautot, Beyond the divide: Boundaries for patterning and stem cell regulation in plants. *Front. Plant Sci.* **6**, 1052 (2015).

47. A. E. Richardson, S. Hake, Drawing a line: Grasses and boundaries. *Plants* **8,** 4 (2018).

48. J. Walsh, M. Freeling, The liguleless2 gene of maize functions during the transition from the vegetative to the reproductive shoot apex. *Plant J.* **19**, 489–495 (1999).

49. J. Moon, H. Candela, S. Hake, The Liguleless narrow mutation affects proximal-distal signaling and leaf growth. *Development* **140**, 405–412 (2013).

50. M. W. Lewis, N. Bolduc, K. Hake, Y. Htike, A. Hay, H. Candela, S. Hake, Gene regulatory interactions at lateral organ boundaries in maize. *Development* **141**, 4590–4597 (2014).

51. L. Wang, H. Yin, Q. Qian, J. Yang, C. Huang, X. Hu, D. Luo, NECK LEAF 1, a GATA type transcription factor, modulates organogenesis by regulating the expression of multiple regulatory genes during reproductive development in rice. *Cell Res.* **19**, 598–611 (2009).

52. R. Johnston, M. Wang, Q. Sun, A. W. Sylvester, S. Hake, M. J. Scanlon, Transcriptomic analyses indicate that maize ligule development recapitulates gene expression patterns that occur during lateral organ initiation. *Plant Cell* **26**, 4718–4732 (2014).

53. K.-i. Hibara, M. R. Karim, S. Takada, K.-i. Taoka, M. Furutani, M. Aida, M. Tasaka, Arabidopsis CUP-SHAPED COTYLEDON3 regulates postembryonic shoot meristem and organ boundary formation. *Plant Cell* **18**, 2946–2957 (2006).

54. D.-K. Lee, M. Geisler, P. S. Springer, LATERAL ORGAN FUSION1 and LATERAL ORGAN FUSION2 function in lateral organ separation and axillary meristem formation in Arabidopsis. *Development* **136**, 2423–2432 (2009).

55. C. Gómez-Mena, R. Sablowski, ARABIDOPSIS THALIANA HOMEOBOX GENE1 establishes the basal boundaries of shoot organs and controls stem growth. *Plant Cell* **20**, 2059–2072 (2008).

56. M. A. Moreno, L. C. Harper, R. W. Krueger, S. L. Dellaporta, M. Freeling, liguleless1 encodes a nuclear-localized protein required for induction of ligules and auricles during maize leaf organogenesis. *Genes Dev.* **11**, 616–628 (1997).

57. W. A. Ricci, Z. Lu, L. Ji, A. P. Marand, C. L. Ethridge, N. G. Murphy, J. M. Noshay, M. Galli, M. K. Mejía-Guerra, M. Colomé-Tatché, F. Johannes, M. J. Rowley, V. G. Corces, J. Zhai, M. J. Scanlon, E. S. Buckler, A. Gallavotti, N. M. Springer, R. J. Schmitz, X. Zhang, Widespread long-range cis-regulatory elements in the maize genome. *Nat. Plants* **5**, 1237–1249 (2019).

58. J. Walsh, C. A. Waters, M. Freeling, The maize gene liguleless2 encodes a basic leucine zipper protein involved in the establishment of the leaf blade-sheath boundary. *Genes Dev.* **12**, 208–218 (1998).

59. K. Houston, A. Druka, N. Bonar, M. Macaulay, U. Lundqvist, J. Franckowiak, M. Morgante, N. Stein, R. Waugh, Analysis of the barley bract suppression gene Trd1. *Theor. Appl. Genet.***125**, 33–45 (2012).

60. Y. Zhao, L. Medrano, K. Ohashi, J. C. Fletcher, H. Yu, H. Sakai, E. M. Meyerowitz, HANABA TARANU is a GATA transcription factor that regulates shoot apical meristem and flower development in Arabidopsis. *Plant Cell* **16**, 2586–2600 (2004).

61. M. R. McKain, H. Tang, J. R. McNeal, S. Ayyampalayam, J. I. Davis, C. W. de Pamphilis, T. J. Givnish, J. C. Pires, D. W. Stevenson, J. H. Leebens-Mack, A phylogenomic assessment of ancient polyploidy and genome evolution across the poales. *Genome Biol. Evol.* **8**, 1150–1164 (2016).

62. J.-W. Wang, R. Schwab, B. Czech, E. Mica, D. Weigel, Dual effects of miR156-targeted *SPL* genes and *CYP78A5/KLUH* on plastochron length and organ size in *Arabidopsis thaliana*. *Plant Cell* **20**, 1231–1243 (2008).

63. K. Miura, M. Ikeda, A. Matsubara, X.-J. Song, M. Ito, K. Asano, M. Matsuoka, H. Kitano, M. Ashikari, OsSPL14 promotes panicle branching and higher grain productivity in rice. *Nat. Genet.* **42**, 545–549 (2010).

64. X. Liang, T. J. Nazarenus, J. M. Stone, Identification of a consensus DNA-binding site for the Arabidopsis thaliana SBP domain transcription factor, AtSPL14, and binding kinetics by surface plasmon resonance. *Biochemistry* **47**, 3645–3653 (2008).

65. C. Li, B. Zhang, MicroRNAs in control of plant development. *J. Cell. Physiol.* **231**, 303–313 (2016).

66. Q. Wang, A. Hasson, S. Rossmann, K. Theres, Divide et impera: Boundaries shape the plant body and initiate new meristems. *New Phytol.* **209**, 485–498 (2016).

67. A. Maugarny-Calès, M. Cortizo, B. Adroher, N. Borrega, B. Gonçalves, G. Brunoud, T. Vernoux, N. Arnaud, P. Laufs, Dissecting the pathways coordinating patterning and growth by plant boundary domains. *PLOS Genet.* **15**, e1007913 (2019).

68. C. Kiecker, A. Lumsden, Compartments and their boundaries in vertebrate brain development. *Nat. Rev. Neurosci.* **6**, 553–564 (2005).

69. P. A. Conklin, J. Strable, S. Li, M. J. Scanlon, On the mechanisms of development in monocot and eudicot leaves. *New Phytol.* **221**, 706–724 (2019).

70. H. Han, X. Liu, Y. Zhou, Transcriptional circuits in control of shoot stem cell homeostasis. *Curr. Opin. Plant Biol.* **53**, 50–56 (2020).

71. A. Goldshmidt, J. P. Alvarez, J. L. Bowman, Y. Eshed, Signals derived from *YABBY* gene activities in organ primordia regulate growth and partitioning of *Arabidopsis* shoot apical meristems. *Plant Cell* **20**, 1217–1230 (2008).

72. B. I. Je, J. Gruel, Y. K. Lee, P. Bommert, E. D. Arevalo, A. L. Eveland, Q. Wu, A. Goldshmidt, R. Meeley, M. Bartlett, M. Komatsu, H. Sakai, H. Jönsson, D. Jackson, Signaling from maize organ primordia via FASCIATED EAR3 regulates stem cell proliferation and yield traits. *Nat. Genet.* **48**, 785–791 (2016).

73. E. Vollbrecht, P. S. Springer, L. Goh, E. S. Buckler IV, R. Martienssen, Architecture of floral branch systems in maize and related grasses. *Nature* **436**, 1119–1126 (2005).

74. E. Bortiri, G. Chuck, E. Vollbrecht, T. Rocheford, R. Martienssen, S. Hake, *ramosa2* encodes a LATERAL ORGAN BOUNDARY domain protein that determines the fate of stem cells in branch meristems of maize. *Plant Cell* **18**, 574–585 (2006).

75. N. Satoh-Nagasawa, N. Nagasawa, S. Malcomber, H. Sakai, D. Jackson, A trehalose metabolic enzyme controls inflorescence architecture in maize. *Nature* **441**, 227–230 (2006).

76. P. Bommert, C. Whipple, Grass inflorescence architecture and meristem determinacy. *Semin. Cell Dev. Biol.* **79**, 37–47 (2018).

77. M. J. Scanlon, K. Ohtsu, M. C. P. Timmermans, P. S. Schnable, Laser microdissection-mediated isolation and in vitro transcriptional amplification of plant RNA. *Curr. Protoc. Mol. Biol.* **87**, 25A.3.1–25A.3.15 (2009).

78. Z. Dong, Y. Xiao, R. Govindarajulu, R. Feil, M. L. Siddoway, T. Nielsen, J. E. Lunn, J. Hawkins, C. Whipple, G. Chuck, The regulatory landscape of a core maize domestication module controlling bud dormancy and growth repression. *Nat. Commun.* **10**, 3810 (2019).

79. L. Conti, D. Bradley, TERMINAL FLOWER1 is a mobile signal controlling Arabidopsis architecture. *Plant Cell* **19**, 767–778 (2007).

80. M. Can, W. Wei, H. Zi, M. Bai, Y. Liu, D. Gao, D. Tu, Y. Bao, L. Wang, S. Chen, X. Zhao, G. Qu, Genome sequence of Kobresia littledalei, the first chromosome-level genome in the family Cyperaceae. *Sci. Data* **7**, 175 (2020).

81. J. Fernandes, Q. Dong, B. Schneider, D. J. Morrow, G.-L. Nan, V. Brendel, V. Walbot, Genome-wide mutagenesis of Zea mays L. using RescueMu transposons. *Genome Biol.* **5**, R82 (2004).

82. S. Knauer, M. Javelle, L. Li, X. Li, X. Ma, K. Wimalanathan, S. Kumari, R. Johnston, S. Leiboff, R. Meeley, P. S. Schnable, D. Ware, C. Lawrence-Dill, J. Yu, G. J. Muehlbauer, M. J. Scanlon, M. C. P. Timmermans, A high-resolution gene expression atlas links dedicated meristem genes to key architectural traits. *Genome Res.* **29**, 1962–1973 (2019).
